# Glypican-1 controls brain size through regulation of fibroblast growth factor signaling in early neurogenesis

**DOI:** 10.1186/1749-8104-4-33

**Published:** 2009-09-04

**Authors:** Yi-Huei Linda Jen, Michele Musacchio, Arthur D Lander

**Affiliations:** 1 Department of Developmental and Cell Biology, Developmental Biology Center and Center for Complex Biological Systems, University of California, Irvine, CA 92697-2300, USA; 2 Department of Molecular Biology and Biochemistry, University of California, Irvine, CA 92697-3900, USA

## Abstract

**Background:**

Cell surface heparan sulfate proteoglycans (HSPGs) act as co-receptors for multiple families of growth factors that regulate animal cell proliferation, differentiation and patterning. Elimination of heparan sulfate during brain development is known to produce severe structural abnormalities. Here we investigate the developmental role played by one particular HSPG, glypican-1 (Gpc1), which is especially abundant on neuronal cell membranes, and is the major HSPG of the adult rodent brain.

**Results:**

Mice with a null mutation in *Gpc1 *were generated and found to be viable and fertile. The major phenotype associated with *Gpc1 *loss is a highly significant reduction in brain size, with only subtle effects on brain patterning (confined to the anterior cerebellum). The brain size difference emerges very early during neurogenesis (between embryonic days 8.5 and 9.5), and remains roughly constant throughout development and adulthood. By examining markers of different signaling pathways, and the differentiation behaviors of cells in the early embryonic brain, we infer that *Gpc1*^-/- ^phenotypes most likely result from a transient reduction in fibroblast growth factor (FGF) signaling. Through the analysis of compound mutants, we provide strong evidence that Fgf17 is the FGF family member through which Gpc1 controls brain size.

**Conclusion:**

These data add to a growing literature that implicates the glypican family of HSPGs in organ size control. They also argue that, among heparan sulfate-dependent signaling molecules, FGFs are disproportionately sensitive to loss of HSPGs. Finally, because heterozygous *Gpc1 *mutant mice were found to have brain sizes half-way between homozygous and wild type, the data imply that endogenous HSPG levels quantitatively control growth factor signaling, a finding that is both novel and relevant to the general question of how the activities of co-receptors are exploited during development.

## Background

Cell surface heparan sulfate proteoglycans (HSPGs) have been implicated as key regulators of patterning and growth in animal development [1,2]. Participation in these events is generally thought to reflect their functions as co-receptors for diverse growth factor families, including fibroblast growth factors (FGFs), Wnts, Hedgehogs and bone morphogenetic proteins (BMPs) [3-6]. In both vertebrates and invertebrates, disruption of heparan sulfate biosynthesis leads to severe, pervasive developmental abnormalities. For example, mice completely deficient in heparan sulfate arrest in gastrulation [7].

In contrast, the elimination of the individual core proteins that carry cell surface heparan sulfate generally produces more subtle or tissue-restricted defects, particularly in mammals [8-16]. Most likely this reflects the relatively large number of cell surface HSPGs (six glypicans and four syndecans in mammals), their overlapping patterns of expression, and a likelihood of functional redundancy that is made particularly high by the fact that carbohydrate moieties mediate much of their function. Despite such complexity, the analysis of core protein mutants has provided novel insights into at least some of the developmental and physiological processes in which HSPGs participate.

The glypicans define a structurally conserved family of glycosylphosphatidylinositol-anchored HSPGs that have been extensively studied for their roles in both development and cancer [17-27]. Of the six glypicans in mammals, glypicans 1 and 2 (Gpc1 and Gpc2) were identified early on as major HSPGs of the developing brain [28-31]. Subsequently, Gpc4 and Gpc5 were shown to be regionally expressed in the developing brain as well [32-34]. Biochemical studies suggest that the most abundant glypican in the rodent brain, at least from late gestation onward, is Gpc1 [29,31]. During development, *Gpc1 *is expressed in both neuroepithelial cells and mature neurons; it is particularly enriched in axons and nerve terminals [35,36]. We reasoned, therefore, that loss of Gpc1 might produce defects in neurogenesis and/or axonal guidance, both of which are driven by growth factors regulated, in many cases, by HSPGs. As described below, the brains of mice rendered null for *Gpc1 *were morphologically normal, except for subtle mispatterning of the anterior cerebellum, but were abnormally small. Unexpectedly, we found that the reduction in brain size in mutant mice reflects a specific role for *Gpc1 *at the earliest stages of neurogenesis, before embryonic day (E)9.5.

## Results

### Generation of glypican-1 null mice

**Figure 1:**
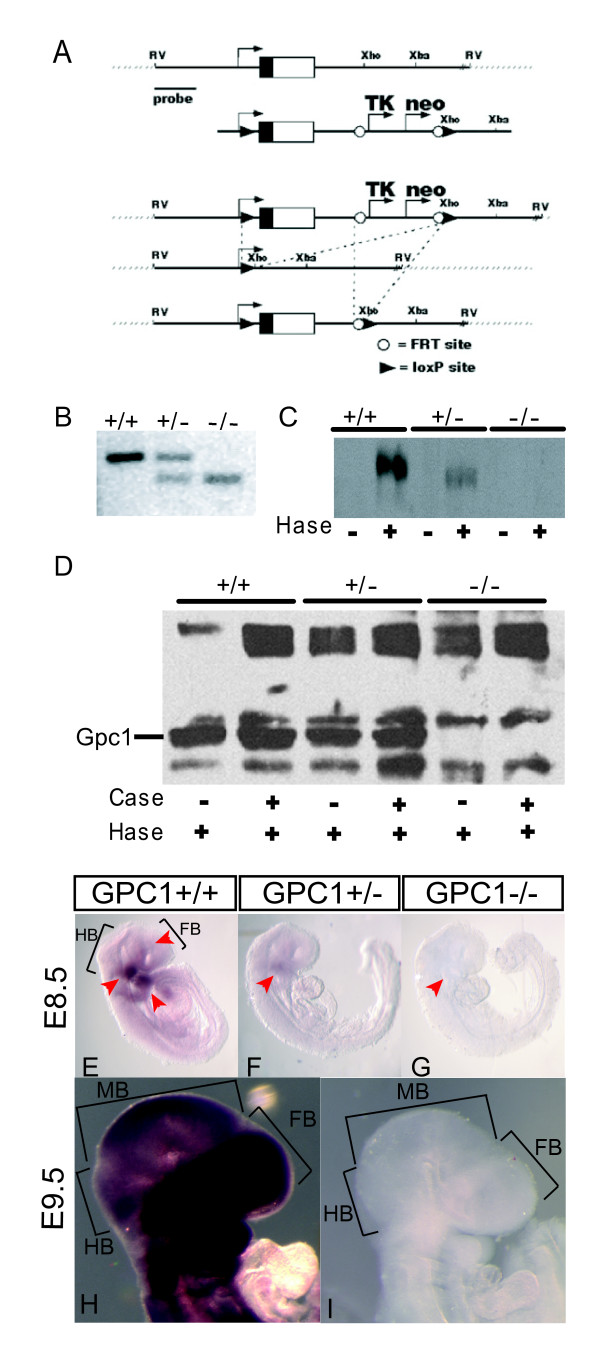
**Targeted mutation of the Gpc1 locus creates a null allele**. **(A) **Targeting strategy. A construct was created in which loxP sites flank exon 1 of *Gpc1 *and FLP recognition target (FRT) sites flank negative (*TK*) and positive (*neo*) selection markers. After transfection into embryonic stem (ES) cells, Southern blotting, using a probe located outside the targeted region, permitted verification of targeted integration. Transient tranfection of targeted ES cells with a Cre expression plasmid was then used to remove *Gpc1 *exon 1 and selection markers, prior to generation of mice. **(B) **Identification of the *Gpc1 *wild-type (+) and mutant (-) allele by PCR in mice. **(C) **Immunoblotting of adult brain membrane fractions with an anti-Gpc1 antibody. The presence of a discrete band found only in samples pretreated with heparitinase (Hase) demonstrates that the immunoreactive molecule is a heparan sulfate proteoglycan (HSPG) core protein. Note the decreased band intensity in heterozygous animals, and complete absence of immunoreactivity in homozygotes. The small apparent difference in mobility and slight tilt of the wild-type band is an artifact of uneven electrophoresis. **(D) **Immunoblotting of adult brain membrane HSPG core proteins using 3G10 antibody. Notice that the loss of a band at the correct molecular weight for Gpc1 in mutant animals is not accompanied by a substantial, consistent change in the presence of other HSPG cores. Hase, Heparitinase; Case, Condroitinase ABC. **(E-G) **Whole-mount *in situ *hybridization for *Gpc1 *at E8.5. Genotypes are as indicated. Red arrowheads point to areas of high Gpc1 expression in the developing brain and branchial arches. Notice that *Gpc1 *expression is greatly reduced or absent in homozygous mutants. Levels in heterozygotes are intermediate. **(H, I) **Whole-mount *in situ *hybridization for *Gpc1 *in wild-type (H) and *Gpc1*^-/- ^(I) embryos at E9.5. Staining has been deliberately overdeveloped to show the absence of signal in the mutant, suggesting that mutant *Gpc1 *mRNA is unstable. FB, forebrain; HB, hindbrain; MB, midbrain.

Homologous recombination was used in embryonic stem (ES) cells to flank the first exon of *Gpc1 *- which includes the translational start site and signal sequence - with loxP sites, and successfully targeted cells were transiently transfected with Cre recombinase to induce excision (Figure 1A). Several independently excised ES clones were injected into C57BL/6 blastocysts to generate chimeric mice, one of which was extensively outcrossed to outbred (CD1; ≥10 generations) and inbred (C57Bl/6; ≥7 generations) mice (Figure 1B). This mutant allele (formally designated *Gpc1*^tm1 Alan^) will be referred to here as *Gpc1*^-^. A second allele - a gene trap insertion of *LacZ *into the fourth intron of Gpc1 - will be referred to as *Gpc1*^*LacZ*^.

*Gpc1*^-/- ^and *Gpc1*^*LacZ*/*LacZ *^mice were viable and fertile on outbred and inbred backgrounds, and appeared grossly normal. Biochemical studies revealed a complete absence of Gpc1 core protein from the brains of homozygous *Gpc1*^-/- ^animals, with intermediate levels in heterozygotes (Figure 1C,D). The expression of other brain heparan sulfate and chondroitin sulfate proteoglycans was not significantly affected (Figure 1D). Immunohistochemistry also demonstrated a loss of Gpc1 staining in both *Gpc1*^-/- ^and *Gpc1*^*LacZ*/*LacZ *^mice (not shown). *In situ *hybridization studies, using both whole embryos and adult brain sections, also revealed a lack of *Gpc1 *transcripts in *Gpc1*^-/- ^animals (Figure 1E-I), suggesting that these animals are essentially protein- and message-null.

### Loss of glypican-1 leads to reduced brain size and subtle patterning abnormalities of the cerebellum

**Figure 2:**
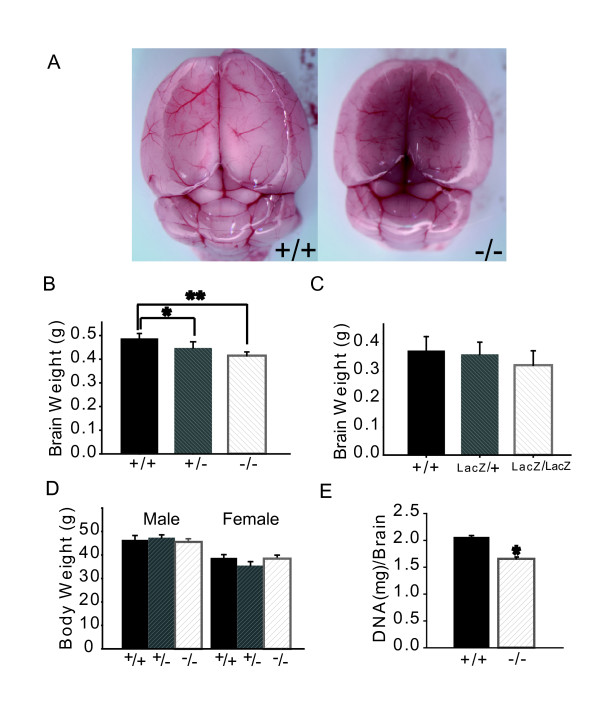
**Adult brain weight from Gpc1 null mutant is significantly reduced**. **(A) **Dorsal views of freshly dissected adult brains. Note the apparent reduction in size of *Gpc1*^-/- ^brains. **(B, C) **Wet weights of fresh adult brains. *Gpc1 *genotypes are as indicated. There is a 15.5% decrease in brain weight in homozygous *Gpc1 *mutants (*P *< 0.0001); heterozygotes show an intermediate phenotype (7.8% decrease; * and **P < 0.0001). (B) N = 32 for +/+; 29 for +/-; 36 for -/-. Reduced brain weight is also observed in *Gpc1*^*LacZ *^mutants. (C) N = 9 for +/+; 10 for *LacZ*/+; 7 for *LacZ*/*LacZ*. **(D) **Body weights of adult *Gpc1 *mutant and wild-type littermates, grouped by sex. No significant effect of genotype is observed. N = 23 for +/+; 30 for +/-; 37 for -/-. **(E) **Total DNA content of mutant and wild-type brains was measured as an indication of cell number. The data imply a 20% reduction in *Gpc1*^-/- ^brains. (*P < 0.001; N = 5 for +/+, and 5 for -/-).

*Gpc1*^-/- ^mice were indistinguishable from wild type in gross appearance, size, lifespan, and a variety of simple behaviors (data not shown). Their internal anatomy appeared normal, with the exception of the brain, which was noticeably small (Figure 2A). On the CD1 background, adult *Gpc1*^-/- ^brains weighed 15.5 ± 1.0% less than wild type, and *Gpc1*^-/+ ^brains weighed 7.8 ± 1.0% less (Figure 2B). Both results were highly statistically significant, as variation in brain size among individuals within mouse strains is normally very small [37].

On a C57/Bl6 background, reductions in *Gpc1*^-/- ^and *Gpc1*^-/+ ^brain weight were also evident, but less pronounced (11% for *Gpc1*^-/-^; data not shown), possibly reflecting the fact that wild-type C57/Bl6 brains are approximately 5% smaller than wild-type CD1 brains to begin with. As shown in Figure 2C, *Gpc1*^*LacZ*/*LacZ *^mice also displayed reduced brain size (the higher variance of these data likely reflects the mixed CD1/C57 background of these animals). The brain weight reduction did not correlate with sex or body weight in Gpc1^- ^or Gpc1^LacZ ^mutant animals (Figure 2D).

To determine whether changes in brain size were due to the presence of fewer cells or smaller cells, DNA was extracted from whole brains and quantified using Hoechst 33258 fluorescence [38]. As shown in Figure 2E, *Gpc1*^-/- ^mice had about 20% less DNA per brain than their wild-type littermates. Thus, loss of *Gpc1 *leads to a 20% decrease in the number of brain cells.

**Figure 3:**
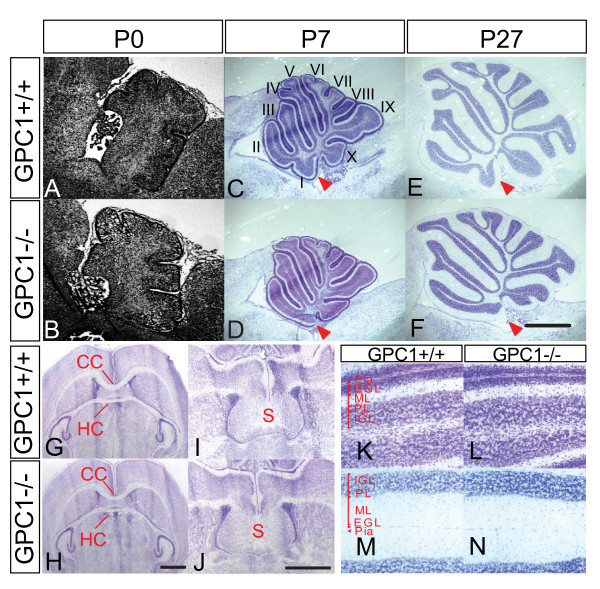
**Cerebellar foliation defects in Gpc1 null mice**. **(A-F) **Development of cerebellar foliation. Nissl-stained mid-sagittal sections are shown at postnatal days (P)0, 7 and 27. Lobes I to X are as marked in (H). As early as P7, a loss or severe reduction in folium one (red arrowhead) can be seen in *Gpc1*^-/- ^mice. **(G-J) **Forebrain anatomy. Coronal sections at the level of the hippocampus (G, H) and septum (I, J) show that midline commissural tracts appear normal in *Gpc1*^-/- ^mice. **(K-N) **Cerebellar lamination. Horizontal sections at P7 (K, L) and P27 (M, N) are shown. The appearance and thickness of the cellular and fiber tract layers are normal in *Gpc1*^-/- ^mice. CC, corpus callosum; EGL, external granular layer; HC, hipocampal commissure; IGL, internal granular layer; ML, molecular layer; PL, purkinje cell layer; S, septum. Bars in (F, H, J) = 1 mm.

Despite this, the shapes of *Gpc1*^-/- ^forebrains were morphometrically normal (Additional file 1), and most structures showed no apparent patterning defects. The major exception was found in the gyri of the cerebellar vermis, where the most anterior lobe (lobe I) either did not form or was rudimentary (Figure 3A-F). In addition, the primary fissure was shortened by 12%, with no significant change in the lengths of the other fissures, or in the overall cerebellar perimeter (data not shown). Notably, structures that are disrupted in heparan sulfate-deficient conditional *Ext1 *mutant mice, such as the interhemispheric commissures and the forebrain septum [39], were present in *Gpc1 *mutants (Figure 3G-J). In addition, no obvious changes in lamination of the cerebellar or cerebral cortices were observed (Figure 3K-N; and data not shown), and staining for markers of neuronal subpopulations, such as calbindin and parvalbumin, appeared normal (data not shown).

### Brain size reduction reflects an early embryonic requirement for *Gpc1*

**Figure 4:**
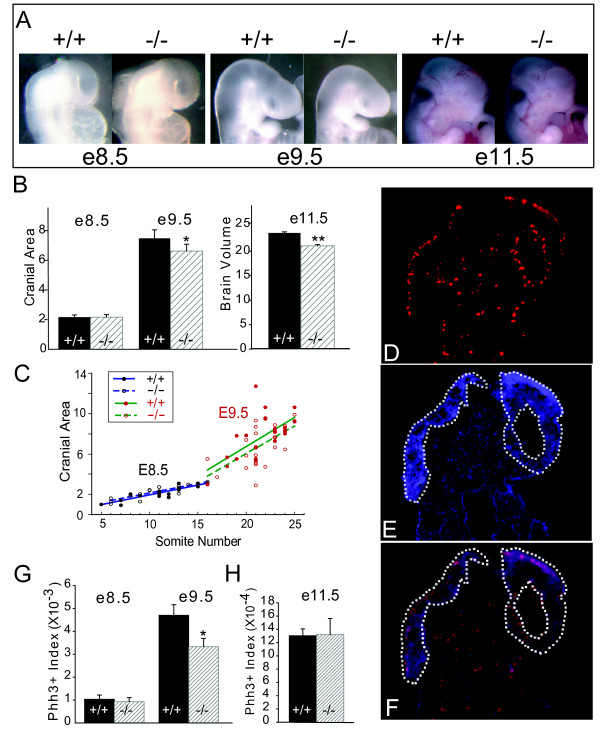
**The *Gpc1 *mutant brain phenotype emerges early in neural development**. **(A) **Lateral images of embryonic day (E)8.5, E9.5 and E11.5 wild-type (+/+) and *Gpc1*^-/- ^(-/-) embryos. Because the developmental stage of embryos is highly variable at E8.5 to E9.5, not every image is fully representative of the average for that genotype (for quantitative data, see (B, C) below). **(B) ***Gpc1*^-/- ^embryos display smaller brains starting at E9.5. Brain size was estimated from photographic images, as described in Additional file 2. Averages of estimated cranial area (at E8.5 and E9.5) and cranial volume (E11.5) were obtained for wild-type and homozygous mutant embryos in multiple litters (N = 23 for +/+ and N = 15 for -/- at E8.5; N = 25 for +/+ and N = 22 for -/- at E9.5; N = 10 for +/+ and N = 18 for -/- at E11.5). Units are pixels^2 ^(× 10^4^) and pixels^3 ^(× 10^6^) for area and volume measurements, respectively. Note the 14.1% decrease in area (* and **P < 0.05) that emerges at E9.5 (consistent with an approximately 20.4% decrease in volume). **(C) **Comparison of cranial area with somite number in E8.5 and E9.5 wild-type (+/+) and *Gpc1*^-/- ^(-/-) embryos. Linear regression lines demonstrate that, even when variation in somite number is controlled for, *Gpc1*^-/- ^brains are of normal size at E8.5, and abnormally small at E9.5. **(D-F) **Quantification of cell proliferation in the embryonic brain. Sagittal sections of E9.5 embryos were immunostained for phosphohistone H3 (PHH3) (D), counterstained with bizbenzamide (E) and the two images merged (F). Note the concentration of labeled cells along the pial and ventricular margins of the neuroepithelium (outlined). **(G, H) **Sets of serial sections were stained in this manner from multiple wild-type and *Gpc1*^-/- ^embryos at E8.5 (N = 5 for both +/+ and -/-), E9.5 (N = 8 for +/+ and N = 7 for -/-), and E11.5 (N = 7 for +/+ and N = 6 for -/-), and numbers of PHH3-labeled cells within the neuroepithelium were counted and normalized to neuroepithelial area for either the entire embryo (G) or the forebrain alone (H). The data show a statistically significant (*P < 0.001) decrease in PHH3-labeling index at E9.5, but no change at E11.5.

Although Gpc1 has been reported to be the major HSPG of the adult brain [35], it is also expressed throughout development of the nervous system, and can be detected in neural tissue as early as head-fold stage (around E7) [35,40]. We therefore measured brain size in *Gpc1*^-/- ^mice at various developmental stages. At birth, *Gpc1*^-/- ^brains weighed 15 to 20% less than wild-type brains, that is, they were smaller to about the same degree as seen in adulthood (data not shown). Prior to birth, weights could not be measured accurately given the small size of embryonic brains and the need to dissect under liquid; instead, volumes were estimated from photographic images of fresh embryos (Additional file 2). As shown in Figure 4A-B the estimated volume of *Gpc1*^-/- ^brains was 22% below that of wild-type littermates as early as E9.5 (Figure 4B). In contrast, brain volumes measured one day earlier (E8.5) exhibited no difference between mutant and wild-type littermate embryos. Apparently, a phenotype of about the same magnitude as seen in adult animals emerges over the course of a single day of development.

To test whether the smaller brain size of E9.5 mutant embryos is a consequence of general developmental delay, we plotted brain size against somite number, a marker of developmental stage (Figure 4C). The data showed a slight trend - which was not statistically significant - toward *Gpc1*^-/- ^embryos being delayed by about 1 somite (at E8.5, wild type = 11.5 ± 2.8 and mutant = 10.3 ± 3.2; at E9.5, wild type = 22.0 ± 2.4 and mutant = 21.2 ± 2.4). Even taking such a trend into account, regression analysis showed that >70% of the brain size difference at E9.5 is independent of somite number (note the downward shift of the regression line at E9.5). These data suggest that, between E8.5 and E9.5, *Gpc1 *plays a specific and important role in nervous system growth.

To test whether that role involves regulation of cell proliferation, we used phospho-histone H3 immunohistochemistry to quantify the density of cells in M-phase (Figure 4D-H). If one considers the early central nervous system as a mass of cycling cells undergoing exponential expansion, with a typical cell cycle time of about 8 hours [41,42], then in order to produce a 20% reduction in cell mass over the course of one day of development (and no further decrease thereafter), one would need to lengthen the cell cycle during that day by about 7%. Thus, in *Gpc1*^-/- ^embryos, we initially expected to see up to an approximately 7% decrease in mitotic labeling index at E8.5, followed by a return to normal on the following day.

Interestingly, we observed a larger and longer-lasting change in labeling index. For example, at E9.5, when we expected proliferation to have returned to wild-type levels, the labeling index in mutant brain neuroepithelium was decreased by 30% (Figure 4G; *P *< 0.001) The effect was specific to the neuroepithelial cells of these embryos (Additional file 3), and did eventually disappear (by E11.5; Figure 4H). We considered the possibility that the larger-than expected drop in proliferation in *Gpc1*^-/- ^embryos was being offset by decreased cell death, but this was not supported by direct measurements. TUNEL staining showed that dying cells are not particularly abundant in the wild-type central nervous system at these stages, and if anything, displayed a trend (not statistically significant) toward being more abundant in the mutant, not less. A likely explanation for the delayed and larger-than-expected drop in neural labeling index became apparent later on, after more information about the probable mechanism of GPC1 action was obtained (see below).

### Redundancy versus compensation within the glypican family

**Figure 5:**
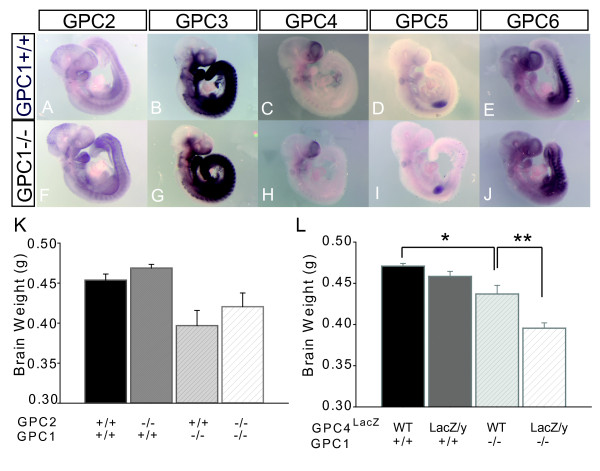
**Influences of other glypicans on brain size regulation**. **(A-J) **Whole-mount *in situ *hybridization for *Gpc2*, *Gpc3*, *Gpc4*, *Gpc5 *and *Gpc6 *in E9.5 wild-type (A-E) and *Gpc1*^-/- ^(F-J) mouse embryos suggests that there is no significant upregulation of other glypicans in response to *Gpc1 *loss. **(K, L) **Wet weights of fresh adult brains from allelic combinations of *Gpc1*^- ^with *Gpc2*^- ^(K) and *Gpc1*^- ^with *Gpc4*^lacZ ^(L). Genotypes are as indicated. The insertion site of the *Gpc4*^LacZ ^gene-trap allele has not been mapped, precluding development of an allele-specific PCR reaction to distinguish *Gpc4*^LacZ/+ ^from *Gpc4*^LacZ/LacZ ^animals. However, since *Gpc4 *is located on the X chromosome, males with a single LacZ allele are unambiguously hemizygous. Thus, the comparison in (L) is between wild-type (both sexes) and LacZ+ males. Note that *Gpc2*^-/- ^brains are not significantly different in size from wild type, nor does loss of *Gpc2 *enhance the phenotype of the *Gpc1*^-/- ^mouse. In contrast, the *Gpc4*^LacZ ^mutation significantly enhances the *Gpc1*^-/- ^phenotype (* and **P < 0.005). N = 7 for *Gpc1*^+/+^;*Gpc2*^+/+^, N = 5 for *Gpc1*^+/+^;*Gpc2*^-/-^, N = 3 for *Gpc1*^-/-^;*Gpc2*^+/+^, N = 3 for *Gpc1*^-/-^;*Gpc2*^-/-^, N = 2 for *Gpc1*^+/+^;*Gpc4*^+/+ ^and N = 4 for Gpc1^+/+^;Gpc4^+/*Y*^, N = 3 for *Gpc1*^-/-^;*Gpc4*^+/+ ^and N = 8 for Gpc1^-/-^;Gpc4^+/*Y*^, N = 14 for *Gpc1*^+/+^;*Gpc4*^Lacz/Y^, N = 9 for *Gpc1*^-/-^;*Gpc4*^LacZ/Y^.

Six structurally similar glypicans are encoded by the mammalian genome. A possible explanation for the relatively mild phenotype in *Gpc1*^-/- ^mutants would be the participation of other glypicans in the regulation of early embryonic neural proliferation. In fact, *Gpc2*, *Gpc*3, *Gpc4*, *Gpc*5, and *Gpc*6 can all be detected in E8.5 and E9.5 embryos, although *Gpc3 *expression is excluded from most of the nervous system, and *Gpc5 *is not strongly expressed in brain until later stages (Figure 5A-E) [30,36,40,43]. Interestingly, the expression levels of Glypicans 2 to 6, as judged by whole mount *in situ *hybridization, were not noticeably altered in *Gpc1*^-/- ^mutants (Figure 5A-J), suggesting that there is little or no compensatory up-regulation of other glypicans in response to loss of *Gpc1*. Levels of *Gpc4 *RNA were also measured by quantitative RT-PCR and were unchanged in *Gpc1*^-/- ^mice (data not shown).

To test for redundancy between *Gpc1 *and the other glypicans that are expressed in early embryonic brain, we generated double mutants with *Gpc2 *and *Gpc4 *(mutants in *Gpc6*, the third glypican that is strongly expressed in early embryonic brain, have not yet been produced). For *Gpc2 *we used a targeted allele that, when homozygous, produces phenotypically normal mice with a complete loss of *Gpc2 *protein (S Saunders and ADL, unpublished data). As shown in Figure 5K, the brains of *Gpc2*^-/- ^mice are not reduced in size, and the brains of *Gpc1*^-/- ^;*Gpc2*^-/- ^double mutants are no smaller than those of *Gpc1*^-/- ^mice.

In contrast, when compound mutants were made between *Gpc1 *and a gene-trap allele of *Gpc4 *(*Gpc4*^*LacZ*^), brain size appeared synergistically reduced (Figure 5L). This experiment may underestimate the contribution of Gpc4, since the *Gpc4*^*LacZ *^allele is very likely not null (unpublished observations). Thus, within the limits of what can be assessed using existing mutants, *Gpc1 *appears to act redundantly with at least *Gpc4 *in controlling brain size.

### Evidence for impairment of FGF signaling in the early *Gpc1 *mutant embryo

Numerous growth factor and morphogen signaling pathways have been implicated in the control of brain growth and development [44-47]. Many of these pathways - including those mediated by Hedgehogs, Wnts, BMPs, and FGFs - are influenced by HSPGs in at least some developmental contexts [1,5,48,49]. To screen for disruption of these pathways in *Gpc1*^-/- ^mice, we performed *in situ *hybridization at E8.5 and E9.5 for known downstream markers or reporter genes.

**Figure 6:**
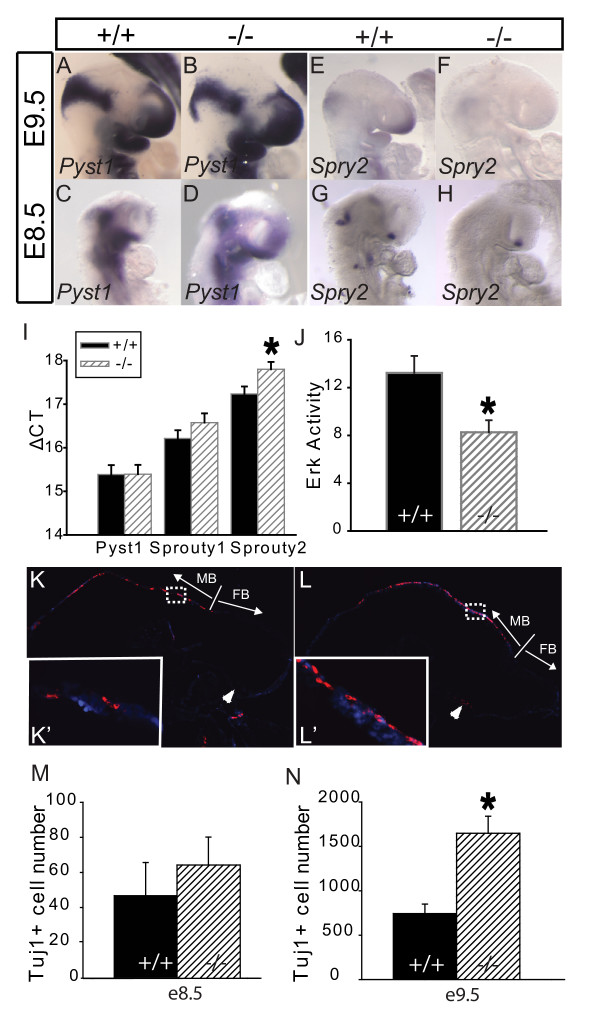
**Loss of Gpc1 results in weakened fibroblast growth factor (FGF) signaling and premature neuronal differentiation in brain**. **(A-D) ***In situ *hybridization for *Pyst1*. **(E-H) ***In situ *hybridization for *Sprouty 2 *(*Spry2*). Both are markers of FGF signaling and only for *Spry2 *was a noticeable change observed between genotypes, with apparent reduced expression in *Gpc1*^-/- ^embryos at both embryonic day (E)8.5 and E9.5. **(I) **Real-time quantitative RT-PCR of *Pyst1*, *Spry1 *(a third FGF-target gene) and *Spry2 *from RNA isolated from E9.5 wild-type and *Gpc1*^-/- ^forebrains. The data imply a significant reduction (32.5%) in *Spry2 *message levels in the *Gpc1 *mutant (*P < 0.01), and a possible reduction in *Spry1*. **(J) **Erk enzymatic activity was measured by *in vitro *phosphorylation in E9.5 dorsal forebrain explants from *Gpc1*^+/+ ^and *Gpc1*^-/- ^embryos. Such explants display a high level of basal (unstimulated) Erk activity, presumably due to the actions of endogenous growth factors. Erk activity was found to be 41% lower in mutant tissue than in wild type (*P < 0.005; units are cpm × 10^4^). **(K, L) **Sagittal sections of E9.5 *Gpc1*^+/+ ^(K) and *Gpc1*^-/- ^(L) embryos were immunostained with Tuj1 (red), a marker for postmitotic neurons, and counterstained with the nuclear marker bizbenzamide (blue). **(K', L') **Enlarged images from white boxes in (K) and (L), corresponding to similar locations in the anterior midbrain. Note the greater density of Tuj1-positive cells in the mutant midbrain. White arrowheads in (K, L) point to the ventral forebrain, where virtually no Tuj1-positive cells are found in the wild-type, but several are present in the Gpc1^-/- ^brain. **(M, N) **Numbers of Tuj1-positive cells within the forebrain (FB) and midbrain (MB) were totaled over serial sections of whole embryos at E8.5 (M) and E9.5 (N). Note the >2-fold increase in Tuj1-positive cells in *Gpc1*^-/- ^brains at E9.5. In contrast, no significant difference is observed at E8.5. At E9.5, N = 7 for +/+ and N = 4 for -/-; at E8.5, N = 4 for each genotype. *P < 0.005.

Although we saw no significant differences between wild-type and mutant embryos at these stages in the expression patterns of marker genes for Sonic hedgehog (Shh), BMP and Wnt signaling (Additional file 4), the data suggested that FGF signaling was potentially attenuated. For one marker of FGF signaling - the mitogen-activated protein (MAP) kinase phosphatase *Pyst1 *(also known as *Mkp3 *and *Dusp6*) - we observed no significant change in the *Gpc1*^-/- ^mouse (Figure 6A-D), but for a second marker - *Sprouty 2 *(*Spry2*) - we noticed a consistent, yet transient, reduction in expression. Specifically, *Spry2 in situ *hybridization was consistently weaker in *Gpc1*^-/- ^brains at E8.5 to E9.5 (Figure 6E-H), but returned to a near-normal level by E10.25 (Additional file 5). To verify this result, we turned to quantitative RT-PCR. As shown in Figure 6I, there was a 40% reduction in *Spry2 *mRNA in *Gpc1*^-/- ^brains at E9.5, with some reduction in *Spry1 *mRNA (also a FGF target gene) as well. In contrast, and in agreement with the data from *in situ *hybridization, *Pyst1 *levels were unchanged.

To examine FGF signaling more directly, we produced short-term E9.5 dorsal forebrain explant cultures from wild-type and *Gpc1*^-/- ^embryos, and measured the enzymatic activity of extracellular regulated kinase (ERK) MAP kinase. As shown in Figure 6J, wild-type explants exhibited a high basal level of ERK activity, suggesting a high degree of endogenous growth factor signaling within the explant. In contrast, *Gpc1*^-/- ^explants exhibited 41% less ERK activity under the same conditions. In response to exogenous FGF2, wild-type explants displayed only a small additional increase in ERK activity, possibly because endogenous signaling was so high. We consistently observed that the exogenous FGF response was even lower in *Gpc1*^-/- ^explants, but due to the small size of the FGF effect we were unable to establish statistical significance for this conclusion (data not shown). Overall, the data strongly suggest that *Gpc1 *deficiency diminishes the response of cells of the early nervous system to FGFs.

### Loss of glypican-1 results in premature differentiation of postmitotic neurons

It is widely reported that FGFs promote the proliferation of neural progenitor cells [50-59]. In most cases, FGFs appear not to act by affecting cell cycle kinetics, but by increasing the probability that the progeny of dividing cells remain in the cell cycle instead of differentiating (that is, FGFs suppress cell cycle exit). Such a mechanism of FGF action has been established, for example, in the cerebral cortex, the midbrain, the olfactory epithelium and the telencephalic subventricular zone [50,52,53,55,58]. Indeed, even in non-neural tissues such as muscle, preventing cell cycle exit seems to be the central mode of action of FGF [60,61].

If impairment of FGF signaling is the primary mechanism by which *Gpc1 *deficiency reduces brain size, we reasoned that *Gpc1*-deficient embryos should exhibit accelerated cell cycle exit and, as a consequence, premature neuronal differentiation. Indeed, this turned out to be the case. As shown in Figure 6K-N, higher than normal numbers of TuJ1+ neurons were detected in the brains of early *Gpc1*^-/- ^embryos. This effect was especially pronounced at E9.5, when there were over twice as many differentiated neurons in the fore- and midbrains of *Gpc1 *mutants as in wild-type animals (*P *< 0.005). To confirm that surplus TuJ1+ neurons were the progeny of cells that had recently undergone cell division, we pulsed embryos at E8.5 with the S-phase label 5-bromo-2'-deoxyuridine (BrdU), and one day later (E9.5) counted the number of TuJ1+/BrdU+ cells in the developing brain. In wild-type embryos we observed an average of 50 ± 3 such cells per mm^2^, whereas in *Gpc1*^-/- ^embryos we observed three times as many (150 ± 49; *P *< 0.05).

Not only do these results support the conclusion that FGF signaling is the main target of Gpc1 in the early central nervous system, they also explain the unexpectedly large, prolonged decreases in mitotic labeling index in *Gpc1 *mutant embryos (Figure 4G). This is because the labeling index represents the ratio of cells in M phase to total cells. When cells leave the cell cycle, they no longer affect the numerator of this ratio, but still contribute to its denominator, thereby causing an alteration in the labeling index that outlasts, for a period of time, any actual disturbance in the proliferative or differentiative behavior of cells (for a quantitative treatment of this point, see the Supplemental Appendix in Additional file 6). In short, the labeling index data in Figure 4G,H correspond well with the expected consequences of a temporary diminution in activity of a factor, such as FGF, that suppresses the differentiation of neuronal progenitors.

### Glypican-1 acts through Fgf17

Several FGF family members have been implicated in brain development, with the subfamily formed by Fgf8, Fgf17 and Fgf18 having been shown to be especially important at early embryonic stages [44,45,47,62-64]. Fgf8, the most intensively studied of the group, plays critical roles in brain patterning and morphogenesis, and it has been suggested that some of the phenotypes in the *Nes-Ext1*^*null *^brain are the result of impaired *Fgf8 *function [39]. However, there is substantial overlap in expression of all three of these Fgfs, which also share similar receptor binding properties, and are all thought to control neural proliferation [65-67].

**Figure 7:**
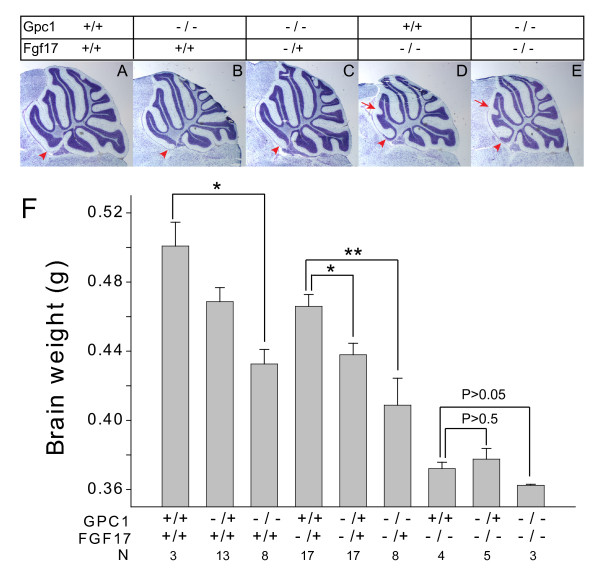
***Gpc1 *phenotypes require *Fgf17***. Compound *Gpc1*/*Fgf17 *genotypes were produced by interbreeding *Gpc1*^+/-^;*Fgf17*^+/- ^mice. **(A-E) **Nissl-stained mid-sagittal sections through the cerebella of adult compound mutants. Red arrowheads mark the anterior-most lobe (lobe I), which disappears in both *Gpc1*^-/- ^and *Fgf17*^-/- ^animals. Red arrows mark the fusion of lobes III and IV, a phenotype observed in *Fgf17*^-/- ^mice. Note that the *Gpc1*^-/-^;*Fgf17*^-/- ^phenotype is no more severe than the single *Fgf17*^-/- ^phenotype. **(F) **Fresh brain weights of adult compound mutants. Genotypes for each category are as indicated; 'N' refers to the number of animals obtained of each genotype. The data show that, on their own, the presence of either one or two copies of mutant alleles for either *Gpc1 *or *Fgf17 *progressively reduces brain size. When animals are null for Fgf17, the presence of mutant *Gpc1 *alleles has no significant effect (*P < 0.005; **P < 0.001; by Student's *t*-test).

In initial studies of compound mutants involving *Gpc1*^- ^and *Fgf8*^*neo *^(a hypomorphic allele of Fgf8), we failed to observe significant genetic interaction (that is, a mutant allele of *Fgf8 *did not further reduce brain weight in a *Gpc1 *mutant; data not shown). In contrast, we were struck by similarities between the reported defects in anterior cerebellar patterning in *Fgf17*^-/- ^mice [66] and those reported here for *Gpc1 *mutants (Figure 3C-F). We therefore generated and analyzed a set of adult *Gpc1*^-^;*Fgf17*^- ^compound mutants (Figure 7).

Representative midline cerebellar morphologies of animals of various compound genotypes are shown in Figure 7A-E. Figure 7F summarizes the distributions of brain weights among the entire collection, by genotype. The data clearly show that, as with *Gpc1*, loss of wild-type *Fgf17 *alleles leads to a progressive reduction in brain size and anterior cerebellar defects. Moreover, in animals that possessed either one or two functional *Fgf17 *alleles, the additional loss of one or two *Gpc1 *alleles led to a further reduction in brain size. However, in animals null for *Fgf17*, loss of *Gpc1 *had no significant phenotypic effect, either on brain size or cerebellar morphology. Complete dependence of the *Gpc1 *phenotype on the presence of a functional *Fgf17 *gene provides strong evidence that Fgf17 is, if not the only FGF through which Gpc1 acts, certainly among the most important, at least with respect to the control of brain size and cerebellar patterning.

## Discussion

The data presented here establish that Gpc1 plays a role in controlling brain size by regulating the behavior of progenitor cells during early brain development. Although the quantitative effect of Gpc1 loss is modest - a 20% decrease in cell number - it is highly significant when compared to normal variation in brain size within genetically homogeneous mice (Figure 2). The effect appears to be due to a shift in the balance of progenitor cell proliferation versus differentiation over the course of approximately one day of development - from E8.5 to E9.5 (Figures 4 and 6). Diminished signaling by FGFs, but not other growth factors, could be demonstrated at around this time period in *Gpc1*^-/- ^mice (Figure 6). Genetic epistasis experiments strongly suggested that reduced Fgf17 signaling accounts for most or all of the *Gpc1 *mutant phenotypes observed here.

The transient and modest effects of *Gpc1 *loss raised the possibility of either compensation by, or redundancy with, other HSPGs, and although no evidence for compensatory up-regulation of other core proteins was obtained, studies with *Gpc1*^-^;*Gpc4*^LacZ ^double mutants suggested that Gpc1 and Gpc4 may have at least partially overlapping functions in controlling brain size (Figure 5). This idea is lent further support by recent studies in *Xenopus*, in which morpholino-mediated knockdown of Gpc4 led to a reduction in size of dorsal forebrain structures [68].

It is noteworthy that *Gpc1*^-/- ^mice failed to display any of the severe developmental phenotypes reported for the *Nestin1-Cre *mediated conditional inactivation of *Ext1*, which produces a mouse in which all heparan sulfate is eliminated from the brain from about E10 onward. The phenotypes of the *Nes1-Ext1*^*null *^mouse include specific hypoplasia of the cerebral hemispheres, absence of the cerebellum and olfactory bulbs, and loss of certain midline commissural tracts. It is possible that the mild deficits in the anterior cerebellum that we observed in the *Gpc1*^-/- ^mouse are related to the cerebellar agenesis seen in the *Nes1-Ext1*^*null *^mouse. Interestingly, even when double mutant mice were made between *Gpc1*^- ^and *Gpc4*^LacZ ^(Figure 5), *Gpc2*^- ^(Figure 5) or *Gpc5*^- ^(YHJ, ADL and S Saunders, unpublished observations), *Nes1-Ext1*^*null *^phenotypes were not observed. It may be that the combined loss of function of multiple glypicans, and/or glypicans as well as syndecans (the other major family of HSPGs), is required to substantially eliminate heparan sulfate function in brain development.

The growth factor families that have been shown to utilize HSPGs as co-receptors include FGFs, BMPs, Hedgehogs and Wnts [1,3,5,44,69-73] - all of which play important roles during early brain development [74-79]. It was surprising, therefore, that evidence only for reduced FGF signaling was obtained in the *Gpc1*^-/- ^mouse. Such evidence included reduced expression of FGF target genes, decreased basal Erk activation, and premature neuronal differentiation (Figure 6), as well as genetic epistasis between *Gpc1 *and *Fgf17 *(Figure 7). Several of the phenotypes in the *Nes1-Ext1*^*null *^mouse are also consistent with reduced FGF signaling, including cerebellar and olfactory bulb agenesis and cerebral cortical hypoplasia [64,66,80,81]. Diminished FGF signaling has also been implicated in the lens phenotypes in *Ndst1 *mutant mice (which produce aberrantly sulfated heparan sulfate [82,83]). These observations suggest that, *in vivo*, FGF signaling may be an especially sensitive indicator of deficits in HSPG function.

A fascinating aspect of the *Gpc1 *mutant phenotype is that the brain weight of heterozygous animals falls halfway between that of wild type and homozygous mutants (Figure 2). This implies that the amount of *Gpc1 *expressed by cells influences, in a continuous fashion, the level of growth factor signaling that cells perceive. In other words, Gpc1 may not merely be necessary for growth factor signaling, but a quantitative regulator of the 'gain' of signaling. Accordingly, regulation of Gpc1 expression may be an important part of the control circuitry that keeps brain size so tightly regulated in mammals [37,84].

Recent work suggests a similar quantitative role - albeit in the opposite direction - for *Gpc3 *[85]. Mouse *Gpc3 *mutants are known to display marked pre- and postnatal overgrowth of many organs, phenocopying Simpson Golabi Behmel syndrome (a syndrome caused by mutations in human GPC3 [8,9,86]). Because *Gpc3 *is on the X chromosome, gene dosage effects are not observable with null alleles, but studies of naturally occurring polymorphisms in the *Gpc3 *regulatory region have recently implied that quantitative variation in the level of *Gpc3 *expression directly (negatively) controls body size [85]. Even though the molecular mechanism of somatic growth inhibition by Gpc3 is likely to differ from the mechanism of brain growth promotion by Gpc1, the implication of both glypicans in quantitative regulation of size is striking. Intriguingly, a variety of studies in *Drosophila *also link invertebrate glypicans to organ size control [87-89].

The present study leaves unresolved the role that glypicans play in axons and nerve terminals, where Gpc1 and Gpc2 are especially abundant [36]. The absence of obvious defects in axonal pathways in the *Gpc1*^-/- ^mouse suggests that these molecules may play more of a role in synaptic physiology than in axonal growth or guidance. Certainly, evidence for the participation of syndecan-2 and syndecan-3 in synapse formation [14,90-92], as well as recent work on the *Drosophila *nervous system [89,93-95], suggests that HSPGs may play a variety of as yet unappreciated roles in basic neurophysiology. To this end, it is intriguing that recent genome-wide association studies in man have identified both GPC1 and FGFR2 (which encodes a major FGF receptor of the brain) as members of a small handful of genetic loci that correlate with risk of schizophrenia [96,97], a psychiatric disorder also associated with a small, but significant, reduction in brain volume [98,99]. Clearly, a detailed behavioral and neurophysiological examination of the *Gpc1 *mutant mouse seems warranted in the future.

## Conclusion

Cell-surface HSPGs are critical for growth and patterning in numerous tissues and organ systems, presumably as a consequence of their actions as growth factor co-receptors. Here we show that Gpc1 controls the size of the mammalian brain through an unexpectedly specific mechanism: regulation of the proliferation/differentiation behavior of progenitor cells during a very early stage of neurogenesis. We provide evidence that this action is mediated through regulation of Fgf17 signaling, and further show that Gpc1's effects are gene-dosage dependent. The data support the view that glypicans, and possibly HSPGs in general, serve as quantitative regulators of the gain of growth factor signaling during neural development.

## Materials and methods

### Mice

*Gpc1*^- ^heterozygous mutants were bred extensively onto CD1 and C57BL/6 backgrounds prior to breeding *inter se *to produce homozygous mutant animals. PCR primers specific for the *Gpc1*^- ^allele were 5'-AGCCGGCTTTTGTTGTCTC-3' and 5'-CACGAGTGTGCTAGGATAGGG-3'. Primers specific for the *Gpc1 *wild-type allele were 5'-CAGCGAAGTCCGCCAGAT-3' and 5'-CAGACCTCCCGAGTGCTAGG-3'.

The following additional mutant alleles were used in this study: gene-trap alleles of *Gpc1 *(GPC1lacZ; Baygenomics ID GST062 San Francisco, CA, USA) and *Gpc4 *(GPC4lacZ; Baygenomics ID Ex194) [100,101]; a targeted null mutation in *Gpc2 *(S Saunders and ADL, unpublished); *Fgf2 *(Jackson Laboratory, Bar Harbor, Maine, USA), a hypomorphic allele of *Fgf8 *(*Fgf8*^*neo *^[102]), *Fgf17 *([74]) and a LacZ-reporter of canonical Wnt signaling (Bat-gal [103]). Wild type CD1 and C57BL/6 mice were from Charles River (San Diego, CA, USA) Genotypes were determined by PCR of tail DNA.

For production of staged embryos, timed matings were used and noon of the day of vaginal plug was considered as E0.5. At early embryonic stages, more precise staging was obtained from somite number. To obtain BrdU-labeled embryos, pregnant mice were injected intraperitoneally with 50 μg of BrdU (B5002; Sigma-Aldrich St. Louis, MO, USA)per gram body weight and embryos were collected 24 hours later.

Mouse colonies were maintained, and all animal experimentation conducted, in accordance with the policies and guidelines of the Institutional Animal Care and Use Committee (IACUC) of the University of California, Irvine. (IACUC protocol number 1998-1656).

### Histology and histochemistry

Adult brains were fast-frozen in 2-methyl-butane prior to cryomicrotome sectioning at 20 μm. Embryos were dissected in cold phosphate-buffered saline (PBS), fixed in 4% paraformaldehyde in PBS at 4°C overnight, cryoprotected in 30% sucrose in PBS at 4°C, and cryomicrotome sectioned at 10 to 20 μm. Sections were stored at -20°C prior to immunohistochemistry or Cresyl-Violet staining. For BrdU staining, cryosections were treated with 2 M HCl for 1 hour at 37°C. Sections were then blocked with 5% goat serum +10% bovine serum albumin/PBS +0.2% Tween20 and incubated with primary antibody diluted in blocking solution at 4°C overnight (anti-Gpc1 [27], 1:500; rabbit anti-phosphohistone H3 (anti-PHH3; Millipore, Billerica, MA, USA, 5 μg/ml, 1:500; Tuj1 (R&D systems, 1:1,000, Minneapolis, MN, USA); anti-BrdU (Abcam, 1:100, Cambridge, MA, USA). Secondary antibodies (alexaFluor goat anti-rabbit IgG, 2 μg/ml alexaFluor goat anti-mouse IgG, 10 μg/ml; Cy3-goat anti mouse, 7 μg/ml (Jackson Immunoresearch, West Grove, PA, USA); or Cy2-goat anti rat, 14 μg/ml (Jackson Immunoresearch)) were applied for 1 hour at room temperature. For quantification of apoptosis, fluorescent TUNEL (terminal deoxynucleotidyl transferase dUTP nick end labeling) assays (Apotag Kit, Serologicals, Norcross, GA, USA) were performed on cyrosections. Hoechst33258 was used at 2 μg/ml for nuclear counterstaining. Fluorescence images were analyzed with a Ziss Axiovert S100 microscope and Hamamatsu C4742-95 digital camera. Wholemount staining for beta-galactosidase activity was performed as described in [104].

### *In situ *hybridization

E8.5-E9.5 embryos, fixed by immersion in 4% paraformaldehyde, were gradually dehydrated in methanol and stored in 100% methanol at -20°C. Wholemount RNA *in situ *hybridization was performed as described [105] with probes synthesized using digoxigenin-labeled NTP mix (Roche, Indianapolis, IN, USA). Probes for glypicans (Glypicans 1 to 6) were obtained by RT-PCR from E13.5 brain total RNA, using the following primer pairs, and subcloned into the PCRII-TOPO vector (Invitrogen, Carlsbad, CA, USA): *Gpc1*, 5'-GCTACATCTCCATCTTCCTTGAC-3' and 5'-AACACACATTATCCACTGACACC-3'; *Gpc2*, 5'-AGTCTGGCGAGGGGTTAGAT-3' and 5'-GGCTACATTGAGGCAGAAGC-3'; *Gpc3*, 5'-GGATGGTGAAAGTGAAGAATCAAC-3' and 5'-GAGAGAAAGAGAAAAGAGGGAAAC-3'; *Gpc4*, 5'-CATGGCACGCTTAGGCTTGCTCGC-3' and 5'-TGGTTGCACTGTTCGCTGACCACG-3'; *Gpc5*, 5'-CGCCAGGATGTTAGTCCATT-3' and 5'-AATTTCTGCCCATTGAGGTG-3'; *Gpc6*, 5'-GCTGTGTATTCTTGCTCTCTCCGGG-3' and 5'-GTACAGCATCCCGTAGGTCCGGAC-3'.

The following additional RNA probes were used: *Pax6 *(335 to 595 bp MN_013627), *Spry2 *(probe used in [106]), *Pyst1 *(probe used in [107]), *Ptc1 *(probe used in [108]), *Msx1 *(*Eco*RI fragment from IMAGE clone 903377). Controls for *in situ *hybridization consisted of sense probes derived from the same DNA fragments.

### Measurement of brain size and DNA content

Postnatal and adult brains were freshly dissected. After removal of olfactory bulbs and remaining spinal cord (at the level of the posterior margin of the cerebellum), brains were immediately weighed on a laboratory scale.

Images of fresh embryos were collected using a Leica MZFLIII stereomicroscope and a SPOT camera (Diagnostic Instruments, Inc. Sterling Heights, MI, USA). For embryos at E11.5 or older, brain height, depth and width were separately measured from lateral and frontal images (Additional file 2), and multiplied to produce a volume estimate. For E8.5 and E9.5 embryos, measurements of area were obtained from perimeter tracings of lateral views using Image J analysis software [109]. At these stages the central nervous system comprises the majority of head tissue, so such tracings included the entire head, stopping ventrally at the rostral border of the first branchial arch, and dorsally at the top of mesencephalon. Volume was then estimated as area^3/2^. In some cases, volume was also estimated by the procedures outlined above for older embryos, and qualitatively similar results were obtained.

DNA content in brain homogenates was measured by enhancement of bisbenzimid fluorescence at 458 nm, as described by Labarca and Paigen [38]. A linear standard curve (1 to 10 μg/ml) was obtained using salmon sperm DNA (Invitrogen).

### Quantitative RT-PCR

Forebrain vesicles of E9.5 and E8.5 wild-type and mutant mice were dissected in ice-cold PBS, and RNA was isolated and column purified (Aurum Total RNA Mini Kit, Bio-Rad, Herculeus, CA, USA) according to the manufacturer's instructions. cDNA was generated by reverse transcription with a mixture of oligo dT and random hexamers (Superscript First-Strand Synthesis kit, Invitrogen). PCR quality controls, experimental runs and statistical methods were performed as described [110,111]. Quantification of total mRNA expression was performed with an Opticon System (MJ Systems CFD-3200, Calgary, Denver, USA) and SYBR-Green (Bio-Rad).

All measurements were normalized to values for 18S RNA in the same samples. All cDNA samples were validated for reverse transcription reaction efficiency and minimal genomic DNA contamination (cDNA/genomic target ratio >10^5^) for 40 cycles in duplicates. Average of duplicated cycle threshold (Ct) values were normalized as ΔCt (Ct_gene of interest _- Ct_reference(18S)_). Relative levels were converted using the 2^-ΔΔCt ^method: ΔΔCt = ΔCt_mutant _- ΔCt_wild-type _[112] Averages of duplicate Ct, normalized ΔCt, ΔΔCt and relative level 2^-ΔΔCt ^and standard errors were calculated using Microsoft Excel.

### Measurement of Erk activity in embryonic explant cultures

E9.5 dorsal telencephalon explants were isolated and cultured as previously described [110]. After 1 hour of incubation at 37°C, FGF2 (R&D Systems) was added at the concentrations indicated for 15 minutes. Explants were briefly washed with 1× PBS and individually homogenized in lysis buffer (1 mM EGTA, 1% Triton X-100, 150 mM NaCl, 50 mM Tris-Cl pH7.4, 1% NP40, 1 μg/ml phenylmethylsulphonyl fluoride (PMSF), 1 μg/ml leupeptin, 1 μg/ml pepstatin, 1 μg/ml aprotinin, 25 μg/ml N-ethylmaleimide (NEM), and phosphatase inhibitors (1 mM NaF, 1 mM Na_3_VO_4_)) with a disposable pestle (Knotes Scientific, Vineland, NJ, USA) Lysed samples were stored at -80°C until use. Erk activity was quantified using an *in vitro *phosphorylation assay (MAP Kinase/Erk Assay kit; Upstate Biotechnology) following the manufacturer's instructions, with or without the 20 μM Erk inhibitor FR180204 (Calbiochem, Gibbstownm, NJ, USA) treatment for 10 minutes prior to the assay. Values in the presence of FR180204 were taken to represent non-ERK phosphorylation activity, and subtracted from each data point. Data were normalized to protein concentration determined by a bicinchoninic acid (BCA) assay [113].

### Subcellular fractionation and analysis of proteoglycan content

Adult brains were dissected in ice-cold PBS and immediately homogenized. to obtain membrane and soluble fractions as described [114]. For SDS-PAGE analysis, samples prepared in this way were digested for 30 minutes at 37°C with Heparinase III or with Heparinase III plus Chondroitinase ABC (all used at 1.5 U/mg of protein; both enzymes were purchased from Seikagaku Corp., Tokyo, Japan) along with a proteinase inhibitor mixture (10 μg/ml pepstatin A, 20 μg/ml leupeptin, 2.5 mg/ml NEM, and PMSF in 50 mM Tris-hydroxyaminomethane, 15 mM phosphoric acid, pH7.3). Digested samples were boiled for 10 minutes in SDS-PAGE sample buffer and loaded at 50 μg protein per lane onto 7.5% SDS-polyacrylamide gels, and subjected to electrophoresis. Gels were transferred to PVDF membrane (Millipore, Billerica, MA, USA) and probed with rabbit anti-glypican-1 (1:3,000) antibody or mouse 3G10 monoclonal antibody (1:2,000; USBiological, Swampscott, MA, USA). Samples without enzyme treatment, or subjected to single enzyme treatment, were used where indicated. Blots were incubated with horseradish peroxidase-conjugated goat anti-rabbit or donkey anti-mouse antibody, as appropriate, and visualized using enhanced chemiluminescence.

## Abbreviations

BMP: bone morphogenetic protein; BrdU: 5-bromo-2'-deoxyuridine; E: embryonic day; ERK: extracellular regulated kinase; ES: embryonic stem; FGF: fibroblast growth factor; Gpc: glypican; HSPG: heparan sulfate proteoglycan; MAP: mitogen-activated protein; NEM: N-ethylmaleimide; *Spry*: *Sprouty*; PBS: phosphate-buffered saline; PHH3: phosphohistone H3; PMSF: phenylmethylsulphonyl fluoride.

## Competing interests

The authors declare that they have no competing interests.

## Authors' contributions

YHJ and ADL conceived of the studies, designed the experiments, interpreted the data and wrote the manuscript. YHJ carried out the experiments, except for the generation of *Gpc1 *knockout mice, which was carried out by MM.

## Supplementary Material

Additional file 1 **Morphometric comparison of wild-type and *Gpc1*^-/- ^forebrains**. Freshly dissected adult brains were photographed from the dorsal surface. Tracings of the outline of the forebrain hemispheres were digitized and each curve converted to a series of points, in intervals of 0.03 radians, on a polar plot centered on its centroid. For each genotype, the curves from different brains were overlayed and rotated so that the medial edges of each tracing (which are relatively straight) were optimally aligned. **(A) **An example from the right hemisphere of nine mutant mice. An average curve was generated by calculating the average distance from the centroid for the family of curves at each angular position. **(B) **Curves representing one standard deviation above and below the average were similarly produced. **(C) **A single curve depicting the ratio of the average mutant and wild-type values at each angular position was then generated, and error bars around this curve were calculated from the square root of the sum of the squares of the relative errors (standard deviation/mean) for the two mutant and wild-type average curves; the comparison of nine mutant and four wild-type right hemispheres using this process is shown. The null hypothesis - that mutant and wild-type forebrains are identical in shape - implies that the ratio curve should be a perfect circle (that is, distance to the centroid for mutant and wild-type should differ by the same proportion at every angular position). As shown in (C), a circle (r = 0.937) fit within the error bars at every angular position, implying that mutant forebrains are approximately 6.3% smaller in linear dimension, with no significant difference in forebrain shape. Note that a 6.3% decrease in linear dimension is consistent with an 18% decrease in volume, comparable to the observed 15.5% decrease in total brain weight (Figure 2). Click here for file

Additional file 2 **Approaches used for estimation of brain volumes of embryos**. Approaches used for estimation of brain volumes of **(A) **E8.5, **(B) **E9.5 and **(C) **E11.5 embryos. Outlines of the primarily neural regions of the head were traced on lateral images of E8.5 to E9.5 embryos, and the enclosed area calculated. At E11.5, separate measurements were made of depth (green line in (C), running from the midbrain-hindbrain boundary (MHB) to the upper nasal-facial junction), height (blue line in (C) connecting the midbrain-forebrain boundary (MFB) to the upper jaw), and width (interocular distance, measured from a frontal view; not shown). Click here for file

Additional file 3 **Evaluation of cell proliferation in wild-type (+/+) and Gpc1 mutant (-/-) embryos at E9.5**. **(A-F) **Sagittal sections of E9.5 embryos were immunostained for phosphohistone H3 (PHH3) (A, D), counterstained with bizbenzamide (B, E) and the two images merged (C,F). As quantified in Figure 4G,H, there are fewer PHH3 labeled cells in the *Gpc1*^-/-^neuroepithelium than in the wild type. **(G) **However, as shown here, the PHH3 labeling index specifically in non-neuroepithelial areas of the head is not significantly different between *Gpc1*^-/- ^and wild-type embryos. Click here for file

Additional file 4 **Lack of apparent difference in Sonic hedgehog, BMP and Wnt signal intensity in *Gpc1*^-/- ^during early stages of brain development**. **(A-J) **Whole mount *in situ *hybridization and reporter gene expression in E8.5 and E9.5 wild type (+/+) and *Gpc1*^-/- ^(-/-) embryos were used to assess levels and distribution of activity of the Hedgehog, BMP and Wnt signaling pathways, all of which have been reported to be influenced by HSPGs. (A-D) *In situ *hybridization for *Patched1*, a marker of Hedgehog signaling. (E-H) *In situ *hybridization for *Msx1*, a marker for BMP signaling. Those small differences in staining intensity that are visible in these images (for example, in the anterior hindbrain) were not consistent findings, but reflect a high degree of embryo-to-embryo variability in *Msx1 *whole mount *in situ *hybridization. (I-J) β-galactosidase activity in embryos crossed onto a BAT-gal background, in which LacZ expression reports canonical Wnt signaling. Click here for file

Additional file 5 **Whole mount *in situ *hybridization for *Spry2 *at E10.25**. When compared with Figure 6E-H, the data suggest that *Spry2 *expression returns to near-normal in *Gpc1*^-/- ^embryos by E10.25. These observations are consistent with the view that the disruption of Fgf signaling in *Gpc1*^-/- ^embryos is transient. Click here for file

Additional file 6 **Supplemental appendix: models for the effect of Gpc1 deficiency on early brain development**. Supplemental appendix: models for the effect of Gpc1 deficiency on early brain development Click here for file
