# Supplementary figures and images for: Glypican-1 controls brain size through regulation of fibroblast growth factor signaling in early neurogenesis
Source: Neural Dev. 2009 Sep 4;4:33. doi: 10.1186/1749-8104-4-33 (PMC2746204; doi:10.1186/1749-8104-4-33)

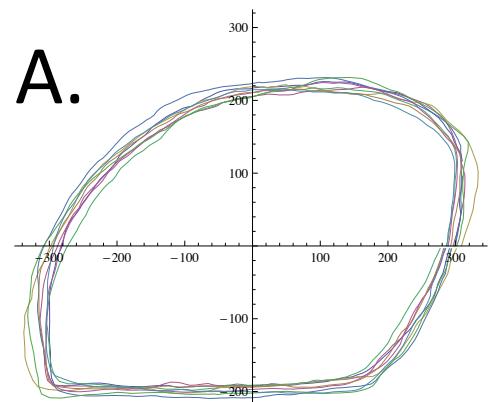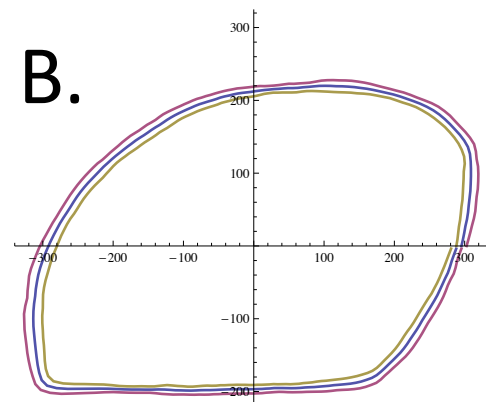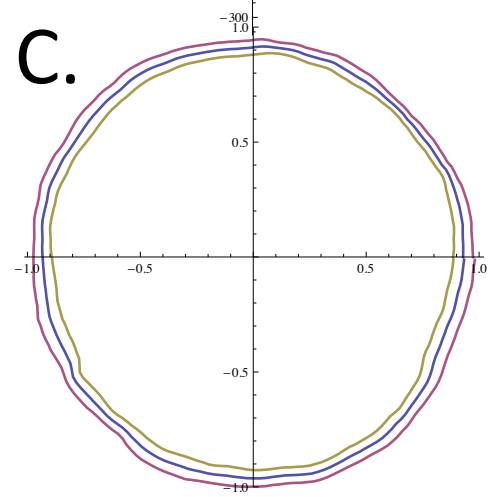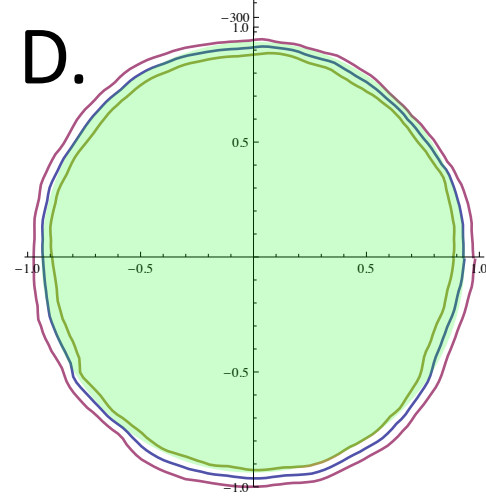

Supplement: Additional file 1 — Morphometric comparison of wild-type and Gpc1-/- forebrains. Freshly dissected adult brains were photographed from the dorsal surface. Tracings of the outline of the forebrain hemispheres were digitized and each curve converted to a series of points, in intervals of 0.03 radians, on a polar plot centered on its centroid. For each genotype, the curves from different brains were overlayed and rotated so that the medial edges of each tracing (which are relatively straight) were optimally aligned. (A) An example from the right hemisphere of nine mutant mice. An average curve was generated by calculating the average distance from the centroid for the family of curves at each angular position. (B) Curves representing one standard deviation above and below the average were similarly produced. (C) A single curve depicting the ratio of the average mutant and wild-type values at each angular position was then generated, and error bars around this curve were calculated from the square root of the sum of the squares of the relative errors (standard deviation/mean) for the two mutant and wild-type average curves; the comparison of nine mutant and four wild-type right hemispheres using this process is shown. The null hypothesis - that mutant and wild-type forebrains are identical in shape - implies that the ratio curve should be a perfect circle (that is, distance to the centroid for mutant and wild-type should differ by the same proportion at every angular position). As shown in (C), a circle (r = 0.937) fit within the error bars at every angular position, implying that mutant forebrains are approximately 6.3% smaller in linear dimension, with no significant difference in forebrain shape. Note that a 6.3% decrease in linear dimension is consistent with an 18% decrease in volume, comparable to the observed 15.5% decrease in total brain weight (Figure 2). [file 1749-8104-4-33-S1.pdf]

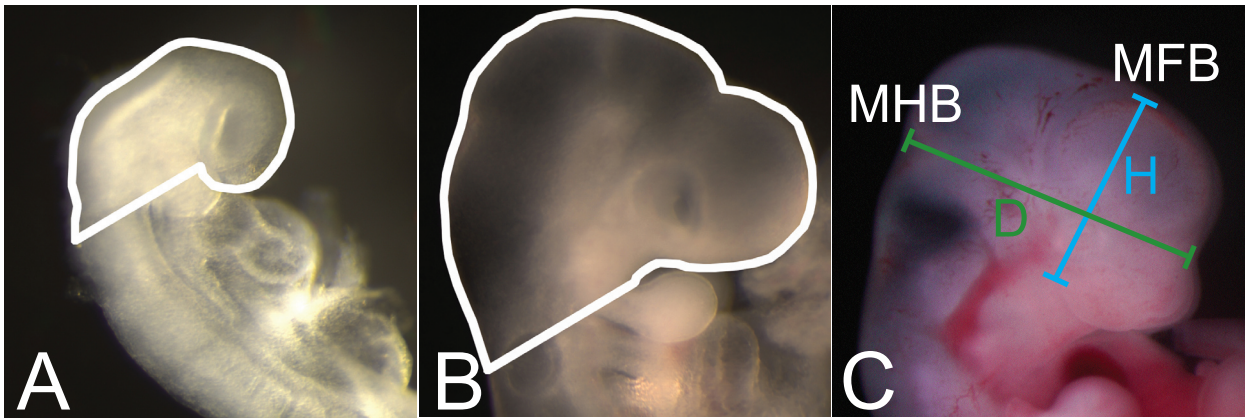

Supplement: Additional file 2 — Approaches used for estimation of brain volumes of embryos. Approaches used for estimation of brain volumes of (A) E8.5, (B) E9.5 and (C) E11.5 embryos. Outlines of the primarily neural regions of the head were traced on lateral images of E8.5 to E9.5 embryos, and the enclosed area calculated. At E11.5, separate measurements were made of depth (green line in (C), running from the midbrain-hindbrain boundary (MHB) to the upper nasal-facial junction), height (blue line in (C) connecting the midbrain-forebrain boundary (MFB) to the upper jaw), and width (interocular distance, measured from a frontal view; not shown). [file 1749-8104-4-33-S2.pdf]

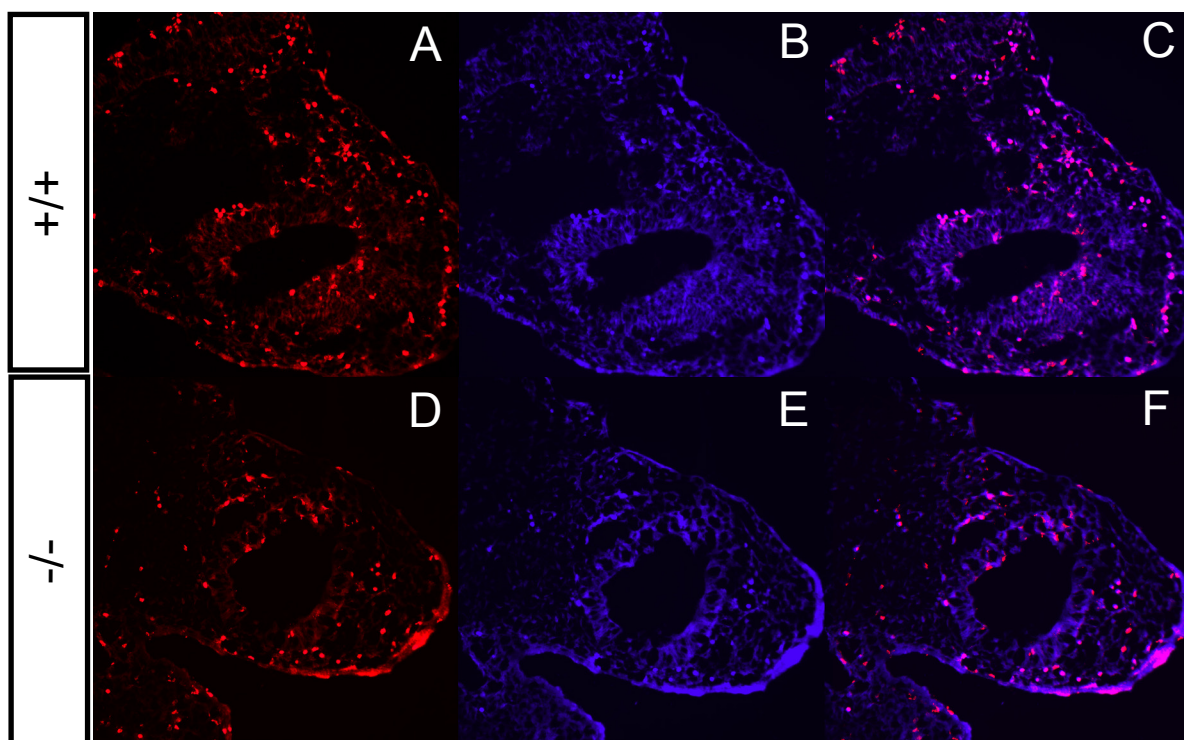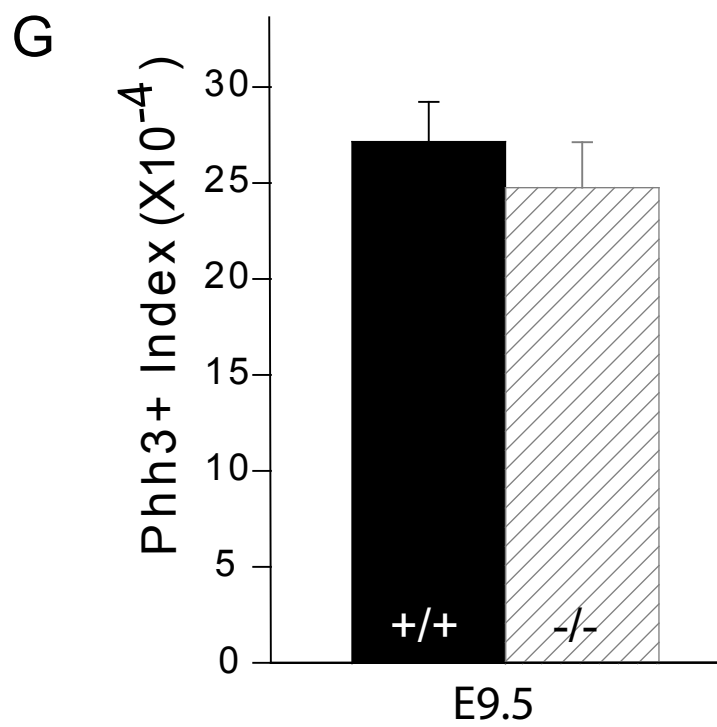

Supplement: Additional file 3 — Evaluation of cell proliferation in wild-type (+/+) and Gpc1 mutant (-/-) embryos at E9.5. (A-F) Sagittal sections of E9.5 embryos were immunostained for phosphohistone H3 (PHH3) (A, D), counterstained with bizbenzamide (B, E) and the two images merged (C,F). As quantified in Figure 4G,H, there are fewer PHH3 labeled cells in the Gpc1-/-neuroepithelium than in the wild type. (G) However, as shown here, the PHH3 labeling index specifically in non-neuroepithelial areas of the head is not significantly different between Gpc1-/- and wild-type embryos. [file 1749-8104-4-33-S3.pdf]

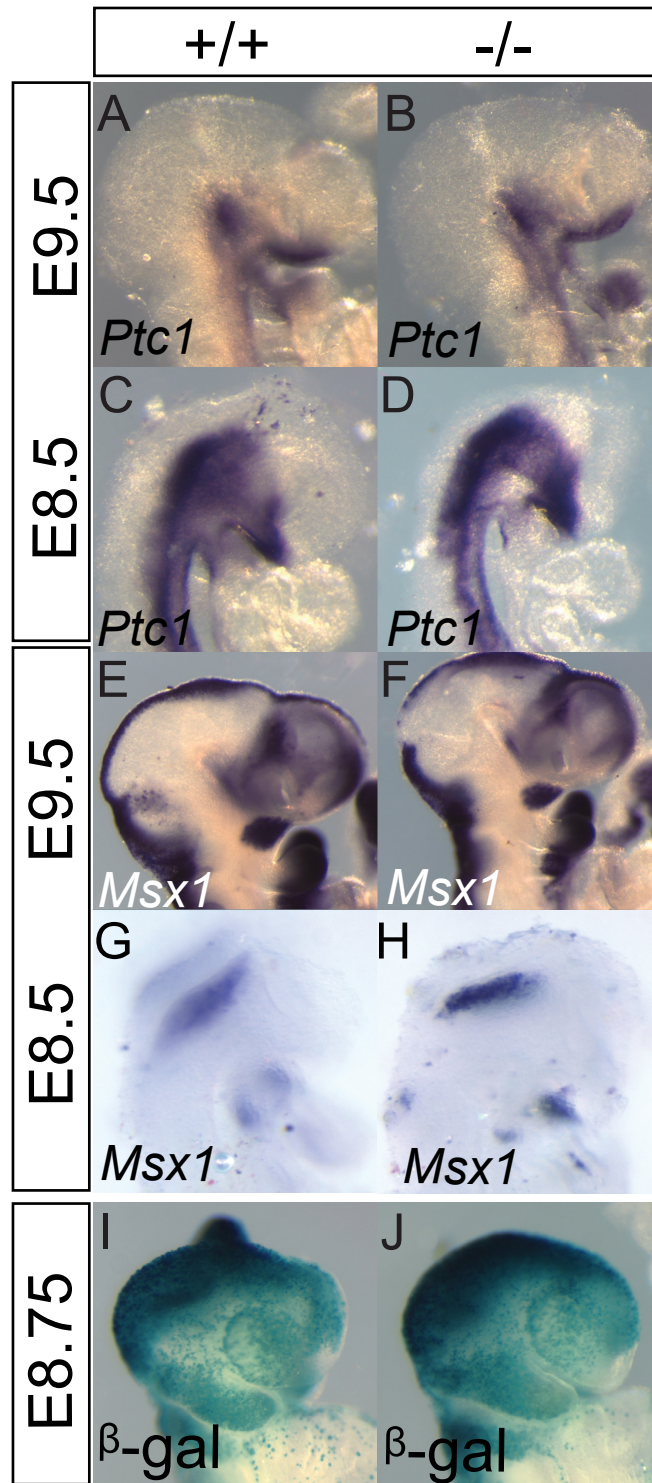

Supplement: Additional file 4 — Lack of apparent difference in Sonic hedgehog, BMP and Wnt signal intensity in Gpc1-/- during early stages of brain development. (A-J) Whole mount in situ hybridization and reporter gene expression in E8.5 and E9.5 wild type (+/+) and Gpc1-/- (-/-) embryos were used to assess levels and distribution of activity of the Hedgehog, BMP and Wnt signaling pathways, all of which have been reported to be influenced by HSPGs. (A-D) In situ hybridization for Patched1, a marker of Hedgehog signaling. (E-H) In situ hybridization for Msx1, a marker for BMP signaling. Those small differences in staining intensity that are visible in these images (for example, in the anterior hindbrain) were not consistent findings, but reflect a high degree of embryo-to-embryo variability in Msx1 whole mount in situ hybridization. (I-J) β-galactosidase activity in embryos crossed onto a BAT-gal background, in which LacZ expression reports canonical Wnt signaling. [file 1749-8104-4-33-S4.pdf]

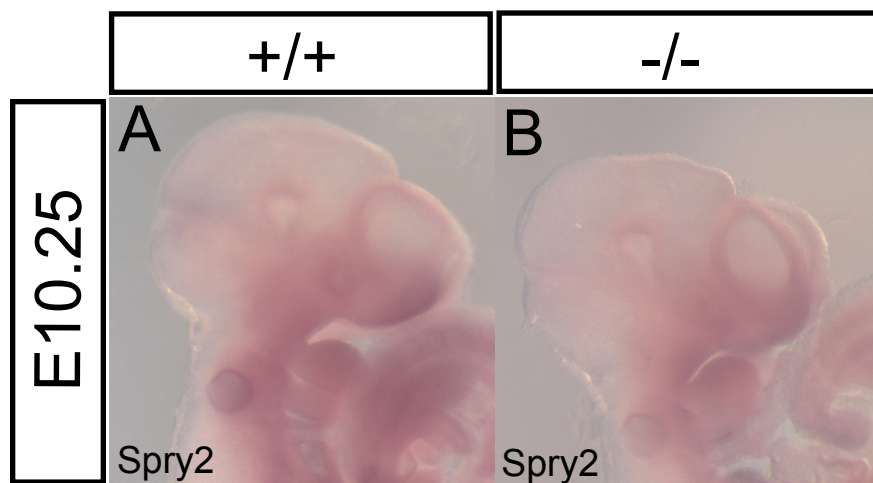

Supplement: Additional file 5 — Whole mount in situ hybridization for Spry2 at E10.25. When compared with Figure 6E-H, the data suggest that Spry2 expression returns to near-normal in Gpc1-/- embryos by E10.25. These observations are consistent with the view that the disruption of Fgf signaling in Gpc1-/- embryos is transient. [file 1749-8104-4-33-S5.pdf]
