# Supplementary material for: Glypican-1 controls brain size through regulation of fibroblast growth factor signaling in early neurogenesis
Source: Neural Dev. 2009 Sep 4;4:33. doi: 10.1186/1749-8104-4-33 (PMC2746204; doi:10.1186/1749-8104-4-33)
Supplement: Additional file 6 — Supplemental appendix: models for the effect of Gpc1 deficiency on early brain development. Supplemental appendix: models for the effect of Gpc1 deficiency on early brain development [file 1749-8104-4-33-S6.pdf]

Models for the effect of glypican-1 deficiency on early brain development

- **Experimental data show that Gpc1- /- mice exhibit normal brain sizes up through embryonic day 8.5 (E8.5), are ~20% smaller than normal at E9.5, and remain roughly 20% smaller than normal thereafter. Thus, some time around E8.5, the loss of Gpc1 leads to a change in the proliferative dynamics of neural progenitor cells. Because the ratio of mutant to wild type brain size drops between E8.5 and E9.5, but not further thereafter, we assume that the effect of loss of Gpc1 is only transient, lasting only about a day (e.g. because the expression of other glypicans becomes adequate to compensate).**

  Here we explore how different mechanisms of action of Gpc1 lead to different expectations for how the phosphohistone H3 labeling index (the percentage of cells in M-phase at any given time) should be affected by the loss of Gpc1.

---

1. A continually cycling population of neural progenitor cells.

- **Here we treat the early nervous system as a uniform population of continually dividing cells undergoing exponential expansion. Let χ[t] represent the number or concentration of cells as a function of time. Let λ be the length of one cell cycle. Then**

$$\chi'[t] == \frac{1}{\lambda}\chi[t];$$

  - **If we use intial conditions of χ[t] = χ0 at t = 0, we may solve this**

$$\text{DSolve}\left[\left\{\chi'[t] == \frac{1}{\lambda}\chi[t],\ \chi[0] == \chi 0\right\},\ \chi[t],\ t\right] \text{ // Simplify // Flatten}$$

$$\{\chi[t] \to e^{t/\lambda}\chi 0\}$$

- **This is the equation for exponential expansion, with a time constant of $1/\lambda$. Loss of Gpc1 could slow this expansion only by increasing λ. The increase in λ would have to be limited to one day in duration (E8.5-E9.5) to account for the resumption of normal expansion after E9.5. at E8.5, λ has been measured at about 8 hours, so Gpc1 would have to act over about 3 cell cycles (Kauffman, 1966, Gressens et al., 1998).**

- **Let us therefore assume that glypican-1 deficiency causes an increase in λ from λ_{wt} to λ_{mut} for three consecutive cell cycles. How many fewer cells will there be at the end of that time? Let λ_{wt}represent the cell cycle length in the wild type, and $\lambda_{mut}$the cell cycle length in the mutant, then:**

$$\frac{e^{t/\lambda_{mut}}\,\chi 0}{e^{t/\lambda_{wt}}\,\chi 0}\ /.\ t \to 3\,\lambda_{wt}\ //\ \text{Simplify}$$

$$e^{-3+\frac{3\,\lambda_{wt}}{\lambda_{mut}}}$$

- **If we let z stand for $\lambda_{mut}/\lambda_{wt}$, we may write this as $e^{-3+\frac{3}{z}}$. To account for a 20 % drop in cell number, then, this expression would need to equal 0.8. Taking logarithms of both sides, and solving, gives us:**

```
Solve[-3 + 3/z == Log[0.8], z] // Simplify // Flatten
{z → 1.08036}
```

- **Thus, an increase in cell cycle length of approximately 8 % for three cell cycles will cause cell number to fall behind by 20 %. In order for cell number not to fall behind any further, cell cycle length must then return to normal.**

  Thus, for a continually cycling population of cells, if we observe a one-day 20% drop in cell number that then stabilizes, we should expect to see an approximately 7.5% (1-1/1.08036) drop in labeling index during that one day, and a return to normal labeling index thereafter. The fact that this is not what we see in the glypican-1 mutant mouse--the drop in labeling index is larger and lasts longer--suggests that modeling the CNS as a continually cycling population of equivalent cells is unrealistic. Below we explore some potentially more realistic models, in which some cells differentiate, and therefore leave the cell cycle, during the time intervals under consideration.

---

2. A simple model for a tissue that reaches a steady state size through continual production of differentiated cells by a stem cell pool, and continual death of the differentiated cells.

- **The following equations model a system in which a dividing cell ("cell type 0"), with a cell cycle time of λ gives rise either to more of itself, or to a postmitotic neuron ("cell type 1"). We take χ_0 and χ_1 to represent the concentration or number of cells of type 0 and 1, respectively. We take *p* to represent the fraction of progeny of divisions of cell type 0 that remain type 0. Thus, 1-*p* is the "leaving fraction", or fraction of progeny of divisions of cell type 0 that differentiate to cell type 1. We take d to represent the rate constant of death of the cell type 1. Since χ_0 , χ_1 and *p* are not necessarily constant, we express them as functions of time, i.e. as χ_0[t] , χ_1[t] and *p*[t]. For further discussion of this approach, see Lander et al., 2009.**

$$\text{sys} = \left\{\chi_0'[t] == \frac{(-1+2\,p[t])\,\chi_0[t]}{\lambda},\ \chi_1'[t] == \frac{2\,(1-p[t])\,\chi_0[t]}{\lambda} - d\,\chi_1[t]\right\}$$

$$\left\{\chi_0'[t] == \frac{(-1+2\,p[t])\,\chi_0[t]}{\lambda},\ \chi_1'[t] == \frac{2\,(1-p[t])\,\chi_0[t]}{\lambda} - d\,\chi_1[t]\right\}$$

- **We simplify the equations by non-dimensionalizing, i.e. we define the unit of time relative to λ. Let τ=t/λ and let δ=d λ, then:**

$$\text{sys1} = \text{sys} \,/.\, d \to \delta/\lambda \,/.\, \left\{\chi_{n\_}'[t] \to \frac{1}{\lambda}\chi_n'[\tau],\ \chi_{n\_}[t] \to \chi_n[\tau],\ p[t] \to p[\tau]\right\} /.\, \lambda \to 1$$

$$\{\chi_0'[\tau] == (-1+2\,p[\tau])\,\chi_0[\tau],\ \chi_1'[\tau] == 2\,(1-p[\tau])\,\chi_0[\tau] - \delta\,\chi_1[\tau]\}$$

- **Let us consider a model in which the major effect of loss of Gpc1 is to decrease p[τ], the replication probability, rather than increase λ, the cell cycle length.**

- **To capture the notion that there is some time interval during which loss of glypican-1 has its effects, we define an interval, from 0<τ< z, and let α represent the value of p[τ] during this interval, and β represent the value of p[τ] afterwards.**

```
sys2 = sys1 /. p[τ] → If[τ < z, α, β]
```

```
{χ0′[τ] == (-1 + 2 If[τ < z, α, β]) χ0[τ], χ1′[τ] == 2 (1 - If[τ < z, α, β]) χ0[τ] - δ χ1[τ]}
```

- **Since glypican - 1 is proposed to increase differentiation (i.e. reduce the value of p[τ]) during the interval 0<τ<z, we model the mutant as having a value of *a* that is lower than that of wildtype, but a value of *b* that is the same.**

```
sys2mut = sys2 /. α → amut;
sys2wt = sys2 /. α → awt;
```

- **Below we simulate how such a system behaves, where the red curves represent the glypican-1 deficient situation. The solid lines in the first graph show total cells; the filled regions show just the differentiated cells. The second graph expresses this as a ratio, i.e. of total cells in mutant to total cells in wildtype.**
  The third graph shows the fraction of cells that are mitotically active, which is what a phosphohistone H3 labeling index would track. The fourth graph shows the ratio of labeling index between mutant and wildtype.

```
params = {z → 3, awt → 0.55, amut → 0.5, β → 0.55, δ → 0.01, endtime → 15};
initconds = {χ0[0] == 1 , χ1[0] == 0.1};
sol1 = Flatten[
   NDSolve[Join[sys2mut /. params, initconds], {χ0[τ], χ1[τ]}, {τ, 0, endtime /. params}]];
sol2 = Flatten[NDSolve[Join[sys2wt /. params, initconds],
     {χ0[τ], χ1[τ]}, {τ, 0, endtime /. params}]];
GraphicsArray[
 {{Plot[Evaluate[{(χ0[τ] + χ1[τ]) /. sol1, (χ0[τ] + χ1[τ]) /. sol2, χ1[τ] /. sol1, χ1[τ] /. sol2}],
     {τ, 0, endtime /. params}, PlotStyle → {Red, Blue, White, White},
     GridLines → {{z /. params}, None}, GridLinesStyle → Dashing[Large],
     Filling → {3 → {Axis, Directive[Opacity[0.1], Red]},
       4 → {Axis, Directive[Opacity[0.1], Blue]}}, AxesLabel → {"time", "cell number"}],
   Plot[Evaluate[{((χ0[τ] + χ1[τ]) /. sol1)/((χ0[τ] + χ1[τ]) /. sol2)}], {τ, 0, endtime /. params}, PlotStyle →
     {Red, Blue}, PlotRange → {0, 1}, AxesLabel → {"time", "mut/wt cell number ratio"},
    GridLinesStyle → Dashing[Large], GridLines → {{z /. params}, {0.8}}]},
  {Plot[Evaluate[{(χ0[τ] / (χ0[τ] + χ1[τ])) /. sol1,
      (χ0[τ] / (χ0[τ] + χ1[τ])) /. sol2}], {τ, 0, endtime /. params}, PlotRange → {0, Automatic},
    PlotStyle → {Red, Blue}, AxesLabel → {"time", "labeling index"},
    GridLinesStyle → Dashing[Large], GridLines → {{z /. params}, None}],
   Plot[Evaluate[{((χ0[τ] / (χ0[τ] + χ1[τ])) /. sol1)/((χ0[τ] / (χ0[τ] + χ1[τ])) /. sol2)}], {τ, 0, endtime /. params},
    PlotStyle → {Red, Blue}, GridLines → {{z /. params}, None},
    GridLinesStyle → Dashing[Large], AxesLabel → {"time", "mut/wt labeling index ratio"}]}}]
```

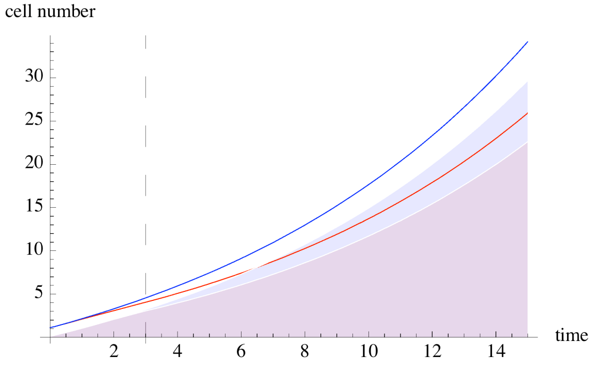

cell number
time

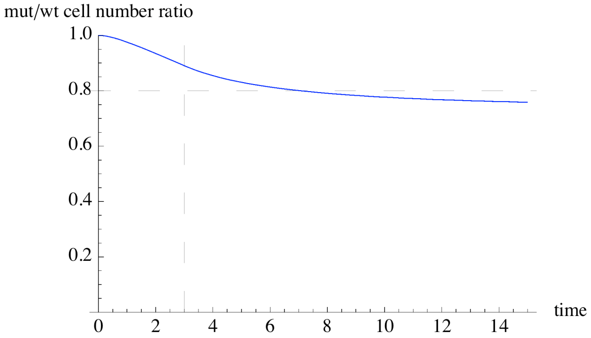

mut/wt cell number ratio
time

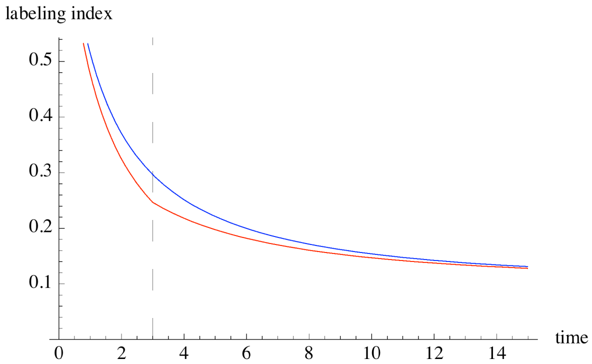

labeling index
time

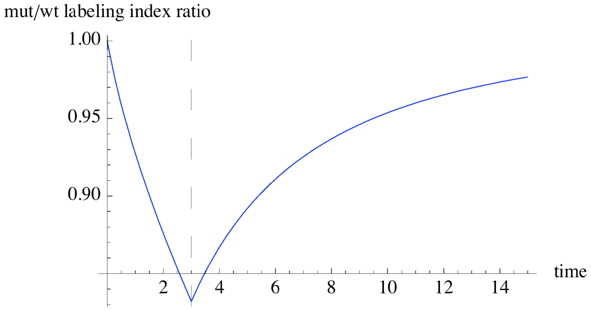

mut/wt labeling index ratio
time

- **Notice how a brief period of lowered p[τ] results in a transient fall in labeling index that peaks at the end of the period of lowered p[τ], and causes a permanent change in tissue size. Although these features better fit the observations for glypican-1 mutants than the previous model, it describes a system that produces differentiated cells at a constrant rate indefinitely. A tissue that undergoes constant cell turnover, such as intestinal epithelium or skin, would be well described by such a model, but in the brain, neurogenesis is confined to a fixed interval, mostly during embryogenesis. We now modify the model to take this into account.**

---

3. A model with a restricted period of neurogenesis

- **The CNS does not undergo neurogenesis at a constant rate. Rather, progenitor cell pools expand and then contract down to very low levels, while at the same time waves of differentiated cells are produced. Nowakowski and colleagues (2002) have pointed out that such behavior naturally arises in a population like that in the last model if the value of p[τ] is initially set to some number >0.5, but then gradually declines to levels below 0.5. When p[τ]>0.5, progenitor cell numbers rise, and when p[τ]<0.5 they contract. Neuron production per progenitor is greatest when p[τ] is smallest, but overall neuron production depends on the number of progenitors in existence. Accordingly, the wave of neuron production peaks as the progenitor cell pool is contracting.**

  For simplicity, we shall model this system by using equations like those in the previous model, but letting p[τ] decline, under wildtype conditions, linearly with time from a starting value of 1 to zero, i.e. p[τ] → 1 - τ/τend, where τend is the time at which p reaches zero.

- **To represent the glypican-1 mutant, we define an interval of time a< τ< b, during which p[τ] is multiplied by *q*, a number less than 1.**

```
sys3 = sys1 /. p[τ] →  (1 - τ / τend) If[a < τ < b, q, 1]
```

$$\left\{\chi_0'[\tau] == \left(-1 + 2\left(1 - \frac{\tau}{\tau\text{end}}\right)\text{If}[a < \tau < b, q, 1]\right)\chi_0[\tau],\right.$$
$$\left.\chi_1'[\tau] == 2\left(1 - \left(1 - \frac{\tau}{\tau\text{end}}\right)\text{If}[a < \tau < b, q, 1]\right)\chi_0[\tau] - \delta\,\chi_1[\tau]\right\}$$

```
sys3mut = sys3;
sys3wt = sys3 /. q → 1;
```

- **Below we simulate how such a system behaves, where the red curves represent the glypican-1 deficient situation. The first graph shows total cells (solid lines) and differentiated cells (filled curves). The second graph expresses the results as a ratio, i.e. of total cells in mutant to total cells in wildtype.**
  The third graph shows the fraction of total cells that is mitotically active, which is what a phosphohistone H3 labeling index would track. The fourth graph shows the ratio of labeling index between mutant and wildtype.

```
params = {q → 0.85, a → 3, b → 5, τend → 11, δ → 0.01};
initconds = {χ0[0] == 1 , χ1[0] == 0};
sol1 = Flatten[
   NDSolve[Join[sys3mut /. params, initconds], {χ0[τ], χ1[τ]}, {τ, 0, τend /. params}]];
sol2 = Flatten[NDSolve[Join[sys3wt /. params, initconds],
    {χ0[τ], χ1[τ]}, {τ, 0, τend /. params}]];
GraphicsArray[{{
   Plot[Evaluate[{(χ0[τ] + χ1[τ]) /. sol1, (χ0[τ] + χ1[τ]) /. sol2, χ1[τ] /. sol1, χ1[τ] /. sol2}
    ], {τ, 0, τend /. params}, PlotStyle → {Red, Blue, White, White},
    GridLines → {{a /. params, b /. params}, None}, GridLinesStyle → Dashing[Large],
    Filling → {3 → {Axis, Directive[Opacity[0.1], Red]},
      4 → {Axis, Directive[Opacity[0.1], Blue]}}, AxesLabel → {"time", "cell number"}],
   Plot[Evaluate[{((χ0[τ] + χ1[τ]) /. sol1) / ((χ0[τ] + χ1[τ]) /. sol2)}
    ], {τ, 0, τend /. params}, PlotRange → {0, 1},
    GridLines → {{a /. params, b /. params}, {0.8}}, GridLinesStyle → Dashing[Large],
    AxesLabel → {"time", "mut/wt cell number ratio"}]},
  {Plot[Evaluate[{χ0[τ] / (χ0[τ] + χ1[τ]) /. sol1, χ0[τ] / (χ0[τ] + χ1[τ]) /. sol2}
    ], {τ, 0, τend /. params}, PlotStyle → {Red, Blue},
    GridLines → {{a /. params, b /. params}, None}, GridLinesStyle → Dashing[Large],
    AxesLabel → {"time", "labeling index"}, PlotRange → {0.2, 0.8}],
   Plot[Evaluate[{((χ0[τ] / (χ0[τ] + χ1[τ])) /. sol1) / ((χ0[τ] / (χ0[τ] + χ1[τ])) /. sol2)}], {τ, 0, τend /. params},
    PlotStyle → {Red, Blue}, PlotRange → {0.7, 1.01},
    GridLines → {{a /. params, b /. params}, None}, GridLinesStyle → Dashing[Large],
    AxesLabel → {"time", "mut/wt labeling index ratio"}]}}]
```

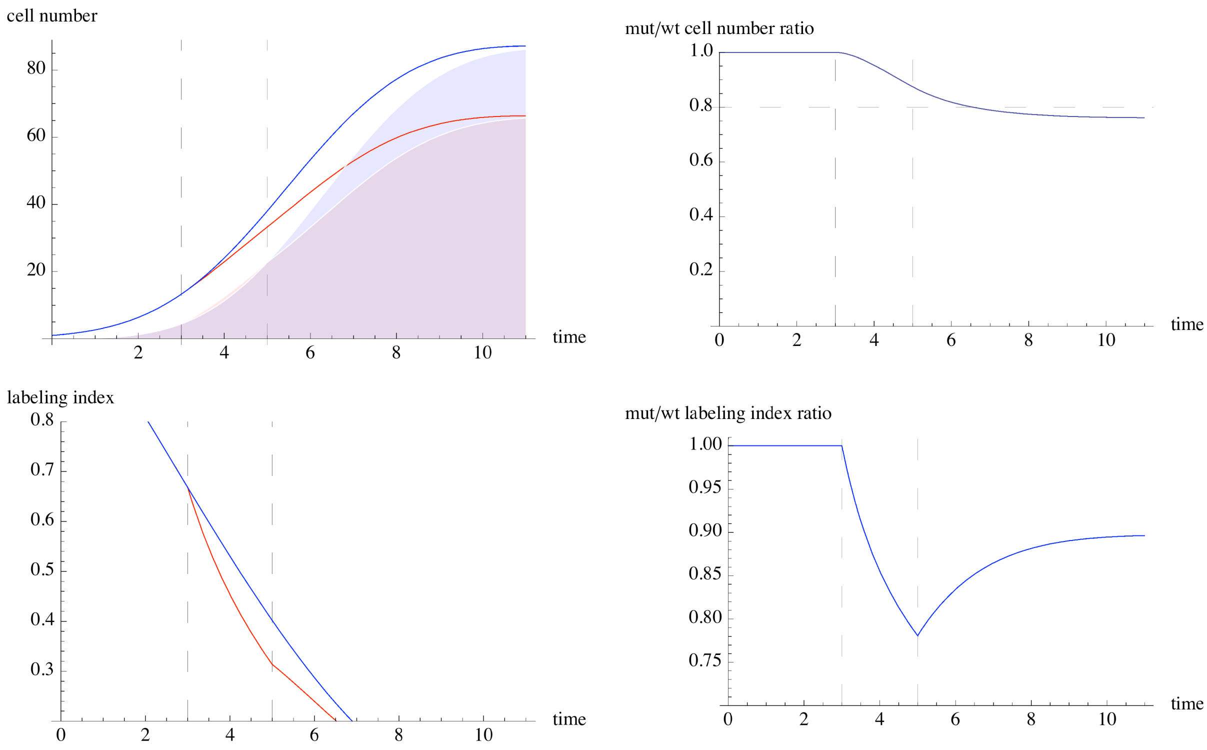

- **As in the previous example, a brief period of lowered p[τ] results in a transient fall in labeling index that peaks at the end of the period of lowered p[τ], and causes a permanent change in tissue size. In the next example, we will consider a case in which proliferation proceeds via two defined lineage stages, with only the second stage being Gpc1 sensitive.**

---

4. A system in which a transit amplifying cell stage, interposed between stem cell and differentiated cell, is the target of glypican-1 action.

- **Now let's consider a system in which there are three cell types, a stem cell, a transit amplifying cell, and a terminally differentiated cell, whose concentrations are represented by χ_0[t], χ_1[t] and χ_2[t] respectively. Let's use λ_0 to represent the length of the cell cycle of the stem cell, and λ_1 for the length of the cell cycle of the transit amplifying cell. Let p_0[t] represent the probability per cell cycle that a stem cell will give rise to a transit amplifying cell, and let p_1[t] represent the probability per cell cycle that a transit amplifying cell will give rise to a terminally differentiated cell.**

$$\text{sys4} = \left\{\chi_0'[t] == \frac{(-1 + 2\, p_0[t])\, \chi_0[t]}{\lambda_0},\right.$$

$$\left.\chi_1'[t] == \frac{2\,(1 - p_0[t])\, \chi_0[t]}{\lambda_0} + \frac{(-1 + 2\, p_1[t])\, \chi_1[t]}{\lambda_1},\ \chi_2'[t] == \frac{2\,(1 - p_1[t])\, \chi_1[t]}{\lambda_1} - d\, \chi_2[t]\right\};$$

- **Define ζ as the ratio λ_1 / λ_2. As in model #2 we can nondimensionalize *d* by defining $\delta = d\lambda_1$, and nondimensionalize time by defining a unit of time $\tau = t / \lambda_1$.**

As in model #3 we can start $p_0[\tau]$at some level>0.5, and let it decline linearly over time.

```
sys4a = sys4 /. {d → δ / λ1, λ0 → λ1 / ζ} /.
    {χn_'[t] → (1/λ1) χn'[τ], χn_[t] → χn[τ], p0[t] → p0[τ], p1[t] → p1[τ]} /. λ1 → 1 /.
  {p0[τ] → p0 (1 - τ / τz), p1[τ] → p1 * If[a < τ < b, q, 1]}
```

$$\left\{\chi_0'[\tau] == \zeta \left(-1 + 2 \left(1 - \frac{\tau}{\tau z}\right) p_0\right) \chi_0[\tau],\right.$$
$$\chi_1'[\tau] == 2 \zeta \left(1 - \left(1 - \frac{\tau}{\tau z}\right) p_0\right) \chi_0[\tau] + (-1 + 2\, \text{If}[a < \tau < b, q, 1]\, p_1)\, \chi_1[\tau],$$
$$\left.\chi_2'[\tau] == 2\, (1 - \text{If}[a < \tau < b, q, 1]\, p_1)\, \chi_1[\tau] - \delta\, \chi_2[\tau]\right\}$$

- **Here $p_1$is the parameter that is affected by glypican deficiency. Again we use q to quantify this effect.**

```
sys4amut = sys4a;
sys4awt = sys4a /. q → 1;
```

- **Note that the calculation of labeling index when there are two proliferating cell types is potentially a little different here, because the precentage of the cell cycle that is S phase or M phase could differ for the two cell types. It is unlikely that this will be a big effect, though, and we will ignore it here.**

```
params = {q → 0.5, a → 10, b → 13, τz → 20, τend → 30, δ → 0.001, p0 → 0.8, p1 → 0.4, ξ → 1};
initconds = {χ0[0] == 1 , χ1[0] == 0, χ2[0] == 0};
sol1 = Flatten[NDSolve[Join[sys4amut /. params, initconds],
    {χ0[τ], χ1[τ], χ2[τ]}, {τ, 0, τend /. params}]];
sol2 = Flatten[NDSolve[Join[sys4awt /. params, initconds],
   {χ0[τ], χ1[τ], χ2[τ]}, {τ, 0, τend /. params}]];
GraphicsArray[{{Plot[Evaluate[{{χ0[τ] + χ1[τ] + χ2[τ]} /. sol1,
      {χ0[τ] + χ1[τ] + χ2[τ]} /. sol2, χ2[τ] /. sol1, χ2[τ] /. sol2}], {τ, 0, τend /. params},
    PlotStyle → {Red, Blue, White, White}, GridLines → {{a /. params, b /. params}, None},
    GridLinesStyle → Dashing[Large], Filling → {3 → {Axis, Directive[Opacity[0.1], Red]},
      4 → {Axis, Directive[Opacity[0.1], Blue]}}, AxesLabel → {"time", "cell number"}],
   Plot[Evaluate[{((χ0[τ] + χ1[τ] + χ2[τ]) /. sol1) / ((χ0[τ] + χ1[τ] + χ2[τ]) /. sol2)}],
    {τ, 0, τend /. params}, PlotStyle → {Red, Blue}, PlotRange → {0, 1},
    GridLines → {{a /. params, b /. params}, {0.8}}, GridLinesStyle → Dashing[Large],
    AxesLabel → {"time", "mut/wt cell number ratio"}]},
  {Plot[Evaluate[{(χ0[τ] + χ1[τ]) / (χ0[τ] + χ1[τ] + χ2[τ]) /. sol1,
      (χ0[τ] + χ1[τ]) / (χ0[τ] + χ1[τ] + χ2[τ]) /. sol2}], {τ, 0, τend /. params},
    PlotStyle → {Red, Blue}, GridLines → {{a /. params, b /. params}, None},
    GridLinesStyle → Dashing[Large], AxesLabel → {"time", "labeling index"}],
   Plot[Evaluate[{ ((χ0[τ] + χ1[τ]) / (χ0[τ] + χ1[τ] + χ2[τ]) /. sol1)
                  / ((χ0[τ] + χ1[τ]) / (χ0[τ] + χ1[τ] + χ2[τ]) /. sol2) }], {τ, 0, τend /. params},
    PlotStyle → {Red, Blue}, GridLines → {{a /. params, b /. params}, None},
    GridLinesStyle → Dashing[Large], AxesLabel → {"time", "mut/wt labeling index ratio"}]}}]
```

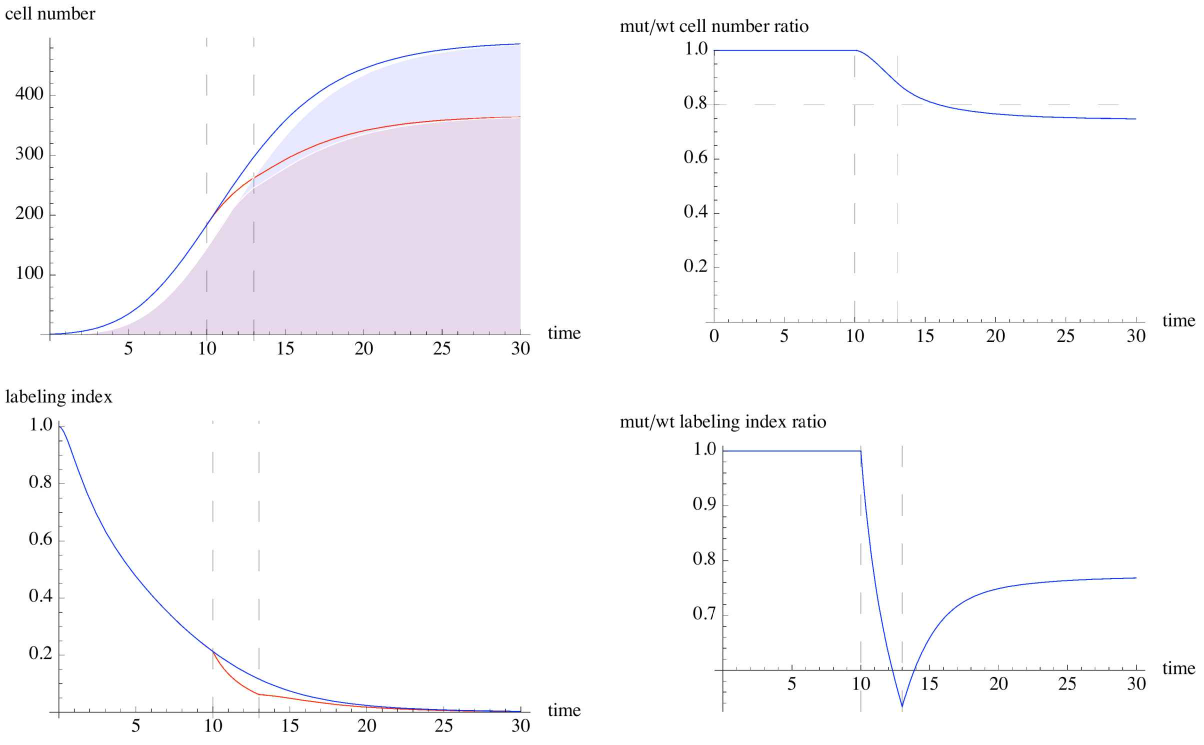

```
params = {q → 0.4, a → 10, b → 13, τz → 30, τend → 40, δ → 0.001, p0 → 0.8, p1 → 0.4, ξ → 1};
initconds = {χ0[0] == 1 , χ1[0] == 0, χ2[0] == 0};
sol1 = Flatten[NDSolve[Join[sys4amut /. params, initconds],
    {χ0[τ], χ1[τ], χ2[τ]}, {τ, 0, τend /. params}]];
sol2 = Flatten[NDSolve[Join[sys4awt /. params, initconds],
   {χ0[τ], χ1[τ], χ2[τ]}, {τ, 0, τend /. params}]];
GraphicsArray[{{Plot[Evaluate[{{χ0[τ] + χ1[τ] + χ2[τ]} /. sol1,
      {χ0[τ] + χ1[τ] + χ2[τ]} /. sol2, χ2[τ] /. sol1, χ2[τ] /. sol2}], {τ, 0, τend /. params},
    PlotStyle → {Red, Blue, White, White}, GridLines → {{a /. params, b /. params}, None},
    GridLinesStyle → Dashing[Large], Filling → {3 → {Axis, Directive[Opacity[0.1], Red]},
      4 → {Axis, Directive[Opacity[0.1], Blue]}}, AxesLabel → {"time", "cell number"}],
   Plot[Evaluate[{((χ0[τ] + χ1[τ] + χ2[τ]) /. sol1) / ((χ0[τ] + χ1[τ] + χ2[τ]) /. sol2)}],
    {τ, 0, τend /. params}, PlotStyle → {Red, Blue}, PlotRange → {0, 1},
    GridLines → {{a /. params, b /. params}, {0.8}}, GridLinesStyle → Dashing[Large],
    AxesLabel → {"time", "mut/wt cell number ratio"}]},
  {Plot[Evaluate[{(χ0[τ] + χ1[τ]) / (χ0[τ] + χ1[τ] + χ2[τ]) /. sol1,
      (χ0[τ] + χ1[τ]) / (χ0[τ] + χ1[τ] + χ2[τ]) /. sol2}], {τ, 0, τend /. params},
    PlotStyle → {Red, Blue}, GridLines → {{a /. params, b /. params}, None},
    GridLinesStyle → Dashing[Large], AxesLabel → {"time", "labeling index"}],
   Plot[Evaluate[{ ((χ0[τ] + χ1[τ]) / (χ0[τ] + χ1[τ] + χ2[τ]) /. sol1) / ((χ0[τ] + χ1[τ]) / (χ0[τ] + χ1[τ] + χ2[τ]) /. sol2) }], {τ, 0, τend /. params},
    PlotStyle → {Red, Blue}, GridLines → {{a /. params, b /. params}, None},
    GridLinesStyle → Dashing[Large], AxesLabel → {"time", "mut/wt labeling index ratio"}]}}]
```

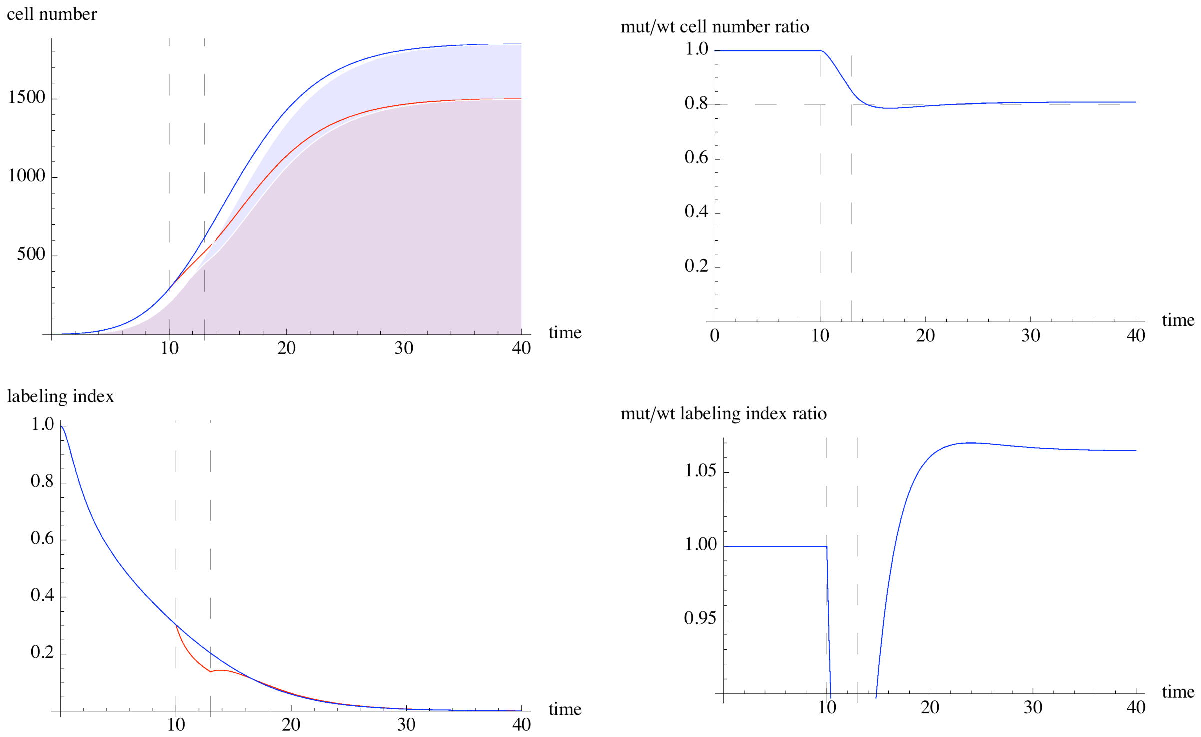

- **What we learn here is that supression of the labeling index is shortlived if most of the mitotic flux is coming from the stem cell, but can be long lived if p0 has declined enough that a lot of the mitotic flux is now coming from the transit amplifying cell.**

Now consider a system with a transit amplifying cell stage, where Gpc1 deficiency affects only the first (stem) cell stage

```
sys5 = sys4 /. {d → δ / λ1, λ0 → λ1 / ζ} /.
    {χn_'[t] → (1/λ1) χn'[τ], χn_[t] → χn[τ], p0[t] → p0[τ], p1[t] → p1[τ]} /. λ1 → 1 /.
  {p0[τ] → p0 (1 - τ / τz) * If[a < τ < b, q, 1], p1[τ] → p1}
```

$$\left\{\chi_0'[\tau] = \zeta \left(-1 + 2 \left(1 - \frac{\tau}{\tau z}\right) \text{If}[a < \tau < b, q, 1]\, p_0\right) \chi_0[\tau],\right.$$

$$\chi_1'[\tau] = 2\,\zeta \left(1 - \left(1 - \frac{\tau}{\tau z}\right) \text{If}[a < \tau < b, q, 1]\, p_0\right) \chi_0[\tau] + (-1 + 2\, p_1)\, \chi_1[\tau],$$

$$\left.\chi_2'[\tau] = 2\, (1 - p_1)\, \chi_1[\tau] - \delta\, \chi_2[\tau]\right\}$$

- **In this case p_0 will be the parameter that is affected by glypican deficiency. Again we use *q* to quantify this effect.**

```
sys5mut = sys5;
sys5wt = sys5 /. q → 1;
```

- **Again the calculation of labeling index when there are two proliferating cell types is potentially a little different here, because the precentage of the cell cycle that is S or M phase could differ for the two cell types. It is unlikely that this will be a big effect, though, and we will again ignore it here.**

```
params = {q → 0.8, a → 10, b → 13, τz → 40, τend → 50, δ → 0.001, p0 → 0.9, p1 → 0.3, ξ → 1};
initconds = {χ0[0] == 1 , χ1[0] == 0, χ2[0] == 0};
sol1 = Flatten[NDSolve[Join[sys5mut /. params, initconds],
    {χ0[τ], χ1[τ], χ2[τ]}, {τ, 0, τend /. params}]];
sol2 = Flatten[NDSolve[Join[sys5wt /. params, initconds],
   {χ0[τ], χ1[τ], χ2[τ]}, {τ, 0, τend /. params}]];
GraphicsArray[{{Plot[Evaluate[{{χ0[τ] + χ1[τ] + χ2[τ]} /. sol1,
      {χ0[τ] + χ1[τ] + χ2[τ]} /. sol2,  χ2[τ] /. sol1, χ2[τ] /. sol2}], {τ, 0, τend /. params},
    PlotStyle → {Red, Blue, White, White}, GridLines → {{a /. params, b /. params}, None},
    GridLinesStyle → Dashing[Large], Filling → {3 → {Axis, Directive[Opacity[0.1], Red]},
      4 → {Axis, Directive[Opacity[0.1], Blue]}}, AxesLabel → {"time", "cell number"}],
   Plot[Evaluate[{((χ0[τ] + χ1[τ] + χ2[τ]) /. sol1) / ((χ0[τ] + χ1[τ] + χ2[τ]) /. sol2)}],
    {τ, 0, τend /. params}, PlotStyle → {Red, Blue}, PlotRange → {0, 1},
    GridLines → {{a /. params, b /. params}, {0.8}}, GridLinesStyle → Dashing[Large],
    AxesLabel → {"time", "mut/wt cell number ratio"}]},
  {Plot[Evaluate[{(χ0[τ] + χ1[τ]) / (χ0[τ] + χ1[τ] + χ2[τ]) /. sol1,
      (χ0[τ] + χ1[τ]) / (χ0[τ] + χ1[τ] + χ2[τ]) /. sol2}
    ], {τ, 0, τend /. params}, PlotStyle → {Red, Blue},
    GridLines → {{a /. params, b /. params}, None},
    GridLinesStyle → Dashing[Large], AxesLabel → {"time", "labeling index"}],
   Plot[Evaluate[{((χ0[τ] + χ1[τ]) / (χ0[τ] + χ1[τ] + χ2[τ]) /. sol1) /
       ((χ0[τ] + χ1[τ]) / (χ0[τ] + χ1[τ] + χ2[τ]) /. sol2)}], {τ, 0, τend /. params},
    PlotStyle → {Red, Blue}, GridLines → {{a /. params, b /. params}, None},
    GridLinesStyle → Dashing[Large], AxesLabel → {"time", "mut/wt labeling index ratio"}]}}]
```

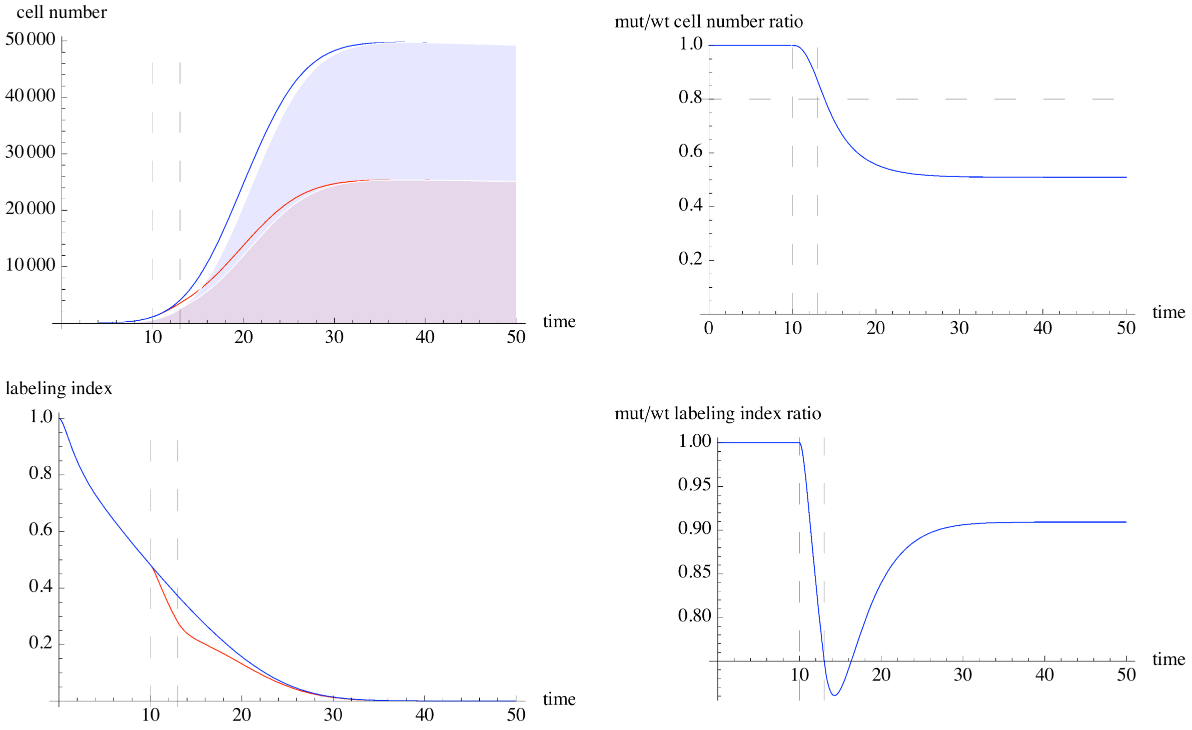

Here we see a change in labeling index that is not only longlasting, but even peaks after that stage at which Gpc1 activity no longer has any influence on proliferation.

```
params = {q → 0.8, a → 2, b → 4, τz → 30, τend → 31, δ → 0.001, p0 → 0.9, p1 → 0.3, ξ → 0.5};
initconds = {χ0[0] == 1 , χ1[0] == 0, χ2[0] == 0};
sol1 = Flatten[NDSolve[Join[sys5mut /. params, initconds],
    {χ0[τ], χ1[τ], χ2[τ]}, {τ, 0, τend /. params}]];
sol2 = Flatten[NDSolve[Join[sys5wt /. params, initconds],
   {χ0[τ], χ1[τ], χ2[τ]}, {τ, 0, τend /. params}]]; GraphicsArray[
 {{Plot[Evaluate[{{χ0[τ] + χ1[τ] + χ2[τ]} /. sol1, {χ0[τ] + χ1[τ] + χ2[τ]} /. sol2, χ2[τ] /. sol1,
      χ2[τ] /. sol2}], {τ, 0, τend /. params}, PlotStyle → {Red, Blue, White, White},
    GridLines → {{a /. params, b /. params}, None}, GridLinesStyle → Dashing[Large], Filling →
     {3 → {Axis, Directive[Opacity[0.1], Red]}, 4 → {Axis, Directive[Opacity[0.1], Blue]}},
    AxesLabel → {"time", "cell number"}, AxesOrigin → {0, 0}],
   Plot[Evaluate[{((χ0[τ] + χ1[τ] + χ2[τ]) /. sol1) / ((χ0[τ] + χ1[τ] + χ2[τ]) /. sol2)}],
    {τ, 0, τend /. params}, PlotStyle → {Red, Blue}, PlotRange → {0, 1},
    GridLines → {{a /. params, b /. params}, {0.8}}, GridLinesStyle → Dashing[Large],
    AxesOrigin → {0, 0}, AxesLabel → {"time", "mut/wt cell number ratio"}]},
  {Plot[Evaluate[{(χ0[τ] + χ1[τ]) / (χ0[τ] + χ1[τ] + χ2[τ]) /. sol1,
      (χ0[τ] + χ1[τ]) / (χ0[τ] + χ1[τ] + χ2[τ]) /. sol2}
    ], {τ, 0, τend /. params}, PlotStyle → {Red, Blue},
    GridLines → {{a /. params, b /. params}, None}, GridLinesStyle → Dashing[Large],
    AxesOrigin → {0, 0}, AxesLabel → {"time", "labeling index"}],
   Plot[Evaluate[{((χ0[τ] + χ1[τ]) / (χ0[τ] + χ1[τ] + χ2[τ]) /. sol1) / ((χ0[τ] + χ1[τ]) / (χ0[τ] + χ1[τ] + χ2[τ]) /. sol2)}],
    {τ, 0, τend /. params}, PlotStyle → {Red, Blue},
    GridLines → {{a /. params, b /. params}, None}, GridLinesStyle → Dashing[Large],
    AxesOrigin → {0, 0}, AxesLabel → {"time", "mut/wt labeling index ratio"}]}}]
```

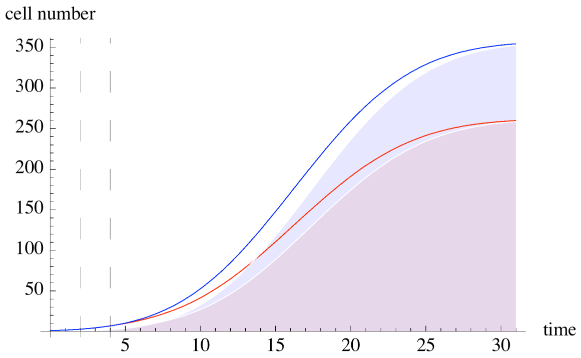

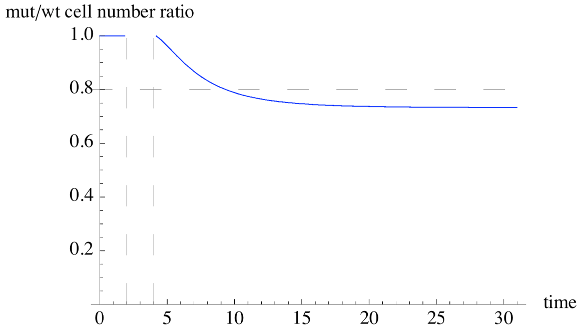

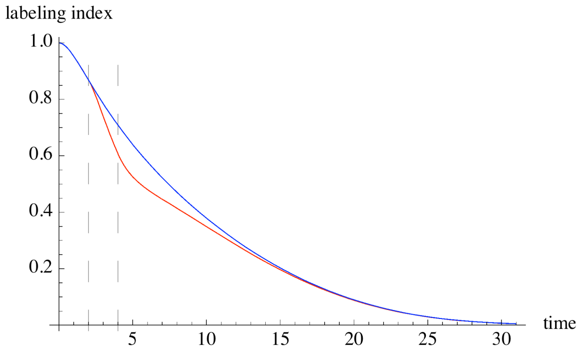

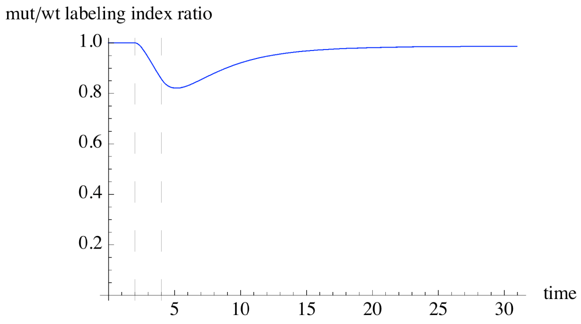

- **In the above case we can clearly see the premature differentiation of cells (red curve transiently**

rises above blue curve--see blowup below)

```
params = {q → 0.8, a → 2, b → 4, τz → 30, τend → 7, δ → 0.001, p0 → 0.9, p1 → 0.3, ξ → 0.5};
initconds = {χ0[0] == 1 , χ1[0] == 0, χ2[0] == 0};
sol1 = Flatten[NDSolve[Join[sys5mut /. params, initconds],
    {χ0[τ], χ1[τ], χ2[τ]}, {τ, 0, τend /. params}]];
sol2 = Flatten[NDSolve[Join[sys5wt /. params, initconds],
   {χ0[τ], χ1[τ], χ2[τ]}, {τ, 0, τend /. params}]]; Plot[Evaluate[
  {{χ0[τ] + χ1[τ] + χ2[τ]} /. sol1, {χ0[τ] + χ1[τ] + χ2[τ]} /. sol2, χ2[τ] /. sol1, χ2[τ] /. sol2}],
 {τ, 0, τend /. params}, PlotStyle → {Red, Blue, Black, Black},
 GridLines → {{a /. params, b /. params}, None}, GridLinesStyle → Dashing[Large], Filling →
  {3 → {Axis, Directive[Opacity[0.1], Red]}, 4 → {Axis, Directive[Opacity[0.1], Blue]}},
 AxesOrigin → {0, 0}, AxesLabel → {"time", "cell number"}]
```

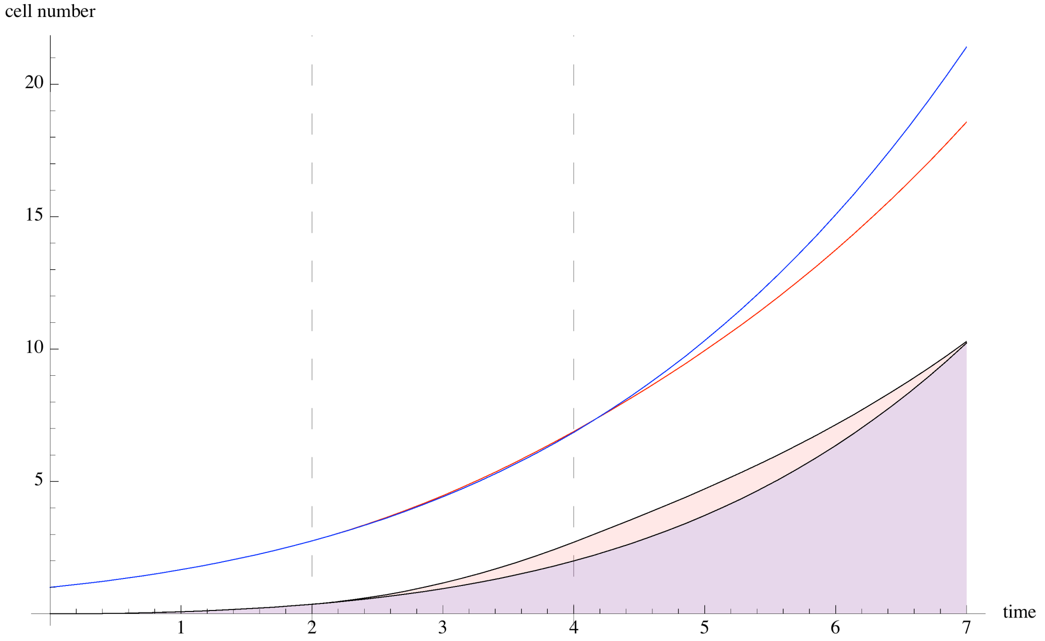

References

- **Nowakowski RS, Caviness VS, Jr., Takahashi T, Hayes NL (2002) Population dynamics during cell proliferation and neuronogenesis in the developing murine neocortex.Results Probl Cell Differ 39 : 1 - 25.**

- **Kauffman SL (1966) An autoradiographic study of the generation cycle in the ten-day mouse embryo neural tube. Exp Cell Res 42: 67-73.**

- **Gressens P, Paindaveine B, Hill JM, Evrard P, Brenneman DE (1998) Vasoactive intestinal peptide shortens both G1 and S phases of neural cell cycle in whole postimplantation cultured mouse embryos. Eur J Neurosci 10: 1734-1742.**

- **Lander AD, Gokoffski KK, Wan FYM, Nie Q, Calof AL (2009) Cell Lineages and the Logic of Proliferative Control. PLoS Biology 7: e15 doi:10.1371/journal.pbio.1000015**
